# Analysing urban and peri-urban youth employment in agribusiness in Malawi

**DOI:** 10.1371/journal.pone.0290877

**Published:** 2023-09-21

**Authors:** Dingase Mkandawire, Sika Gbegbelegbe, Justus V. Nsenga, Mastewal Yami, Edwin Kenamu

**Affiliations:** 1 College of Social Science and Humanities, Sokoine University of Agriculture (SUA), Morogoro, Tanzania; 2 International Institute of Tropical Agriculture (IITA), Chitedze Research Station, Lilongwe, Malawi; 3 Department of Development Studies, Sokoine University of Agriculture (SUA), Morogoro, Tanzania; 4 Independent Consultant, Addis Ababa, Ethiopia; 5 Georg-August-Universität Göttingen, Göttingen, Germany; University of Sargodha, PAKISTAN

## Abstract

The study aims to analyse key determinants of urban and peri-urban youth employment in agribusiness in Malawi to support youth policies. A mixed-methods approach is used, which combines both quantitative and qualitative analyses. The quantitative method involved a Bivariate Logit Model and Multinomial Logit Model to analyse nationally representative survey data from the Fourth Integrated Household Survey in 2016–2017. The qualitative method employed thematic analysis to data generated through Focus Group Discussions and Key Informant Interviews for key stakeholders involved in agri-business in Lilongwe district. The qualitative analysis, which focuses on a case study for urban and peri-urban youth in agribusiness, was used to validate, and provide context for the quantitative analysis. The results revealed that a majority of the urban and peri-urban youth engaged in agribusiness across Malawi work in sole farming (family farms or ganyu); in addition, women outnumber men in terms of engagement in agribusiness, and this stems from tradition. In addition, the determinants that affect youth’s engagement in agribusiness consist of demographic factors, institutional support, assets, and shocks. It was also shown that men were more likely than women to be engaged in sole farming, but they were as likely as women to be engaged in other forms of agribusiness. The policy recommendation from this study is that programs aimed at supporting youth engagement in agribusiness should consider a variety of factors; If resources are limited, the programs should ensure that they offer capacity strengthening for the youth in the form of extension services and practical training in agribusiness.

## 1 Introduction

Youth in low-income countries, including countries in sub-Saharan Africa (SSA), struggle to secure gratifying livelihoods as they enter the labour force in astounding numbers [1]. In addition, employment in the formal wage sector in sub-Saharan Africa remains elusive [2], with many youths not able to get employment in the formal sector [3]. Most often, youth work in small, informal family businesses where they are self-employed or where they work without pay [4]. According to the International Labour Office [5], in 2019, 23 percent of youth globally were engaged in formal employment while 77 percent were engaged in informal employment. It is projected that over 4.8 million young Africans will enter the labour force each year between 2010 and 2050 [6]. Moreover, the evidence shows that youth in Africa are three times as likely as older adults to be unemployed [7].

In Malawi, youth unemployment is high standing at 27.5 percent for youth aged 15–24 years and 23.0 percent for youth aged 15–34 years compared to 20.4 percent for the total unemployment rate [8]. According to Fox et al. [2], the employment structure in African countries, Malawi included, has not changed much due to their failure to structurally transform into high-income economies with more productive agricultural and non-agricultural sectors. The efforts to address youth unemployment in Malawi have been inadequate with most policies and programs not paying explicit attention to the agricultural sector [9, 10].

Agriculture can be a source of livelihood for youth in Malawi. According to FAO [11], agriculture in Malawi remains the principal livelihood opportunity for many young people, as agriculture is seemingly the default source of livelihood for most, including those living in urban centres [12]. Agriculture is also a source of livelihood for youth in Africa. As such, some agribusiness interventions across Africa have produced favourable outcomes such as youth startups and youth employment [13]. The Government of Malawi and development partners have been implementing youth-based programs in agribusiness with the aim of providing youth with training and resources to promote employment in agribusiness. The Integrated Youth Development Programme (IYDP), One Village One Product (OVOP) program in Malawi and Associated Centre for Agro-Based Development and Entrepreneurship Support (ACADES) are some of the programs.

In some cases, the employment opportunities for young people in agriculture have not materialized in gainful employment due to various constraints. Some of the constraints for African youth include lack access to credit and improved technologies; limited practical skills, and limited access to fair markets as well as lack of access to land [14, 15]. These challenges are due to limited inclusion and the lack of a favourable environment to create a sense of ownership by the youth engaged in agricultural value chains [16]. Indeed, in Malawi, the youth and agricultural policy framework provides little support to youth in terms of access to affordable farm inputs, land, extension services, even value addition initiatives and access to markets [9]. Evidence shows that youth in Malawi face various and interconnected challenges; as a result, they suffer simultaneous well-being deprivations [17]. According to Ismail [18], there is limited knowledge on employment experiences and barriers for some youth groups, including urban and rural youth. In this study, we examine the factors that influence youth employment in agribusiness in the urban and peri-urban areas of Malawi.

Some studies have analysed the determinants of employment in agribusiness [12, 19–21]. For example, Gelan et al. [21], using a linear regression model, found that education, farm income, livestock ownership and access to credit, inputs, and land influence employment in agriculture in Ethiopia. Byishimo et al. [22] found that land inheritance had a negative influence on youth migration and non-agricultural based employment in Rwanda.

In Malawi, Van den Broeck et al. [19], using linear probability models, found that demographic factors, shocks, and job characteristics are important determinants of off-farm employment among the youth. In addition, Benson et al. [12] used a multinomial logit regression and found that education, age, gender, dependency ratio, education, ethnicity, distance to market and shocks (drought) influence youth’s employment in agriculture and/or in industry/services for Malawi. Kafle et al. [20] examined the dynamics of youth employment in Malawi and Tanzania and found that a high degree of youth in Malawi are in farming while their participation in agri-food enterprises remained constant between 2010 and 2013 (around 15%). In addition, youth’s participation in different employment options in Malawi was driven by push/negative factors such as the loss of jobs or livelihood options; it could also be driven by pull/opportunity factors such as the higher opportunity costs of remaining in agriculture.

Most studies have focused on rural settings and little research has been done on factors that influence youth employment in agribusiness in the urban areas of Malawi. This study aims to contribute to addressing the knowledge gap on the determinants of urban and peri-urban youth employment in agribusiness by using the nationally-representative survey data generated as part of the World Bank Living Standards Measurement Study–Integrated Household Survey (LSMS-IHS) initiative. The study also employs a triangulation approach which complements the quantitative analysis with a qualitative analysis, thereby enabling an in-depth understanding of the key determinants of urban and peri-urban youth employment in agribusiness. The main objective of this study was to identify key factors influencing urban and peri-urban youth employment in agribusiness in Malawi. Specific objectives were as follows:

- Identify the determinants of youth employment in agricultural-related enterprises in urban and peri-urban
- Analyse opportunities and challenges experienced by urban and peri-urban youth in agricultural-related enterprises

This study is organized as follows. Section 2 describes the data and methods used for the analysis. Section 3 presents results. Section 4 presents a discussion of the findings. Section 5 presents the conclusions and implications of the findings.

## 2 Materials and methods

The study used a mixed method approach which combines quantitative and qualitative methods. The quantitative method consisted of econometric regressions on a nationally representative sample of youth engaged in agribusiness in the urban and peri-urban areas of Malawi. The qualitative method employed thematic analysis with qualitative data obtained from key stakeholders involved in youth in agriculture, including those related to the Associated Centre for Agro-Based Development and Entrepreneurship Support (ACADES). ACADES operates in Lilongwe and Mchinji districts and started in the year 2013. It has grown to be the largest network of youth in agribusiness in Malawi with over 3000 members; it provides the much-needed evidence as it promotes employment through investment in agribusiness by supporting youth in agribusiness. The qualitative analysis which consisted of a case study provided validation and context for some of the quantitative results. The combination of both methods should strengthen policy recommendations.

### 2.1 Sampling technique and data collection

#### 2.1.1 Quantitative data: Secondary data (survey)

**Fig 1:**
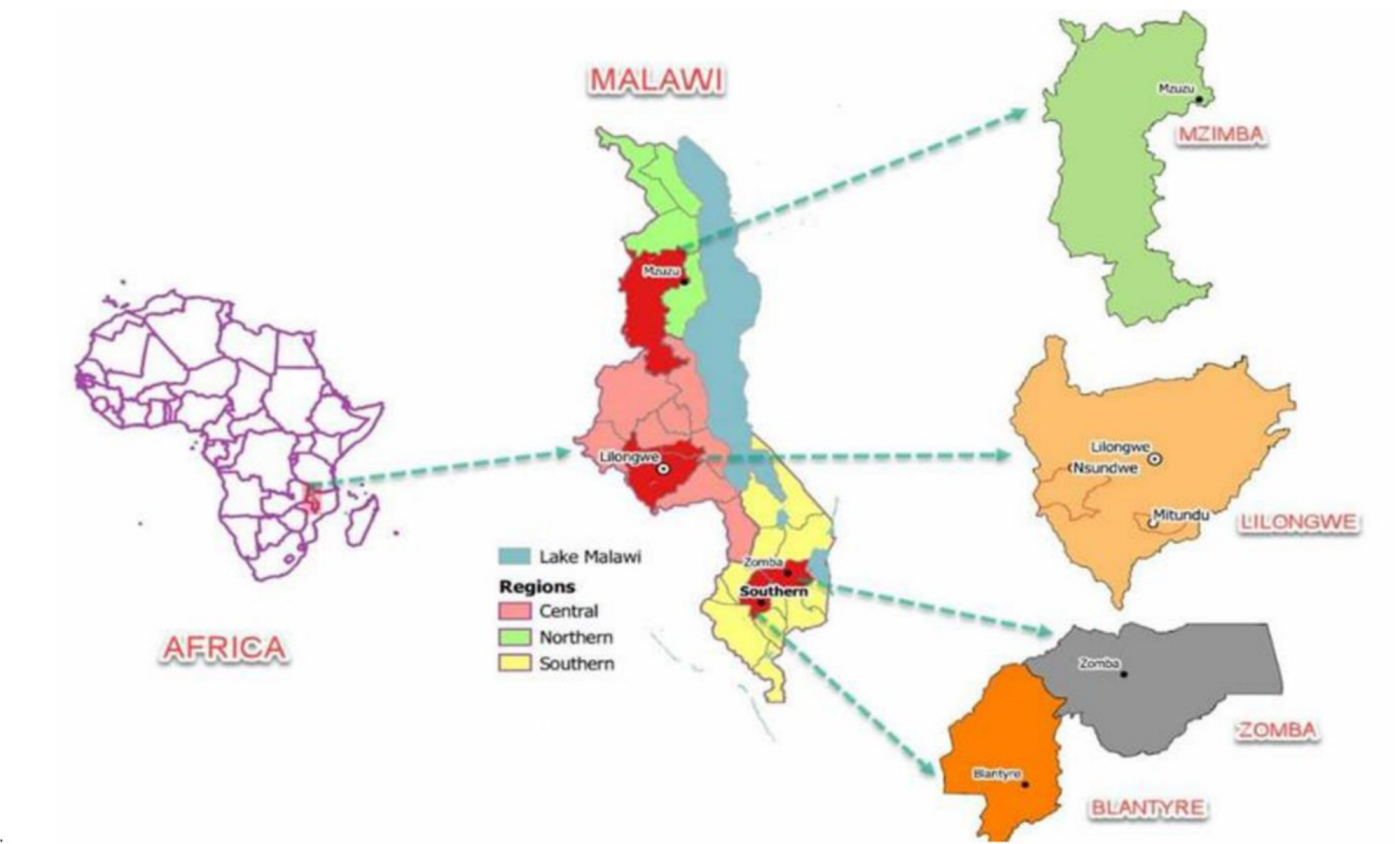
Map showing the four urban and peri-urban areas in Malawi. **Source:** Authors’ construct with support from Clyde Kalima.

Quantitative data was obtained from the nationally representative Fourth Integrated Household Survey (IHS4) data for Malawi, generated as part of the World Bank’s Living Standards Measurement Integrated Survey in Agriculture (LSMS-ISA) initiative. The analysis relied on survey data of 2016–17 and involved a nationally representative sample of youth aged 15–34 years who resided in four urban and peri-urban areas of Malawi, namely, Blantyre, Lilongwe, Mzuzu and Zomba. The final sample consisted of youth engaged in agribusiness or unemployed; youth who were still in school and thus unemployed were removed from the sample (**Fig 1**).

#### 2.1.2 Qualitative data

Qualitative data were generated from focus group discussions (FGDs) and key informant interviews (KIIs) in Lilongwe district; the district houses Lilongwe city, the capital city of the country (Fig 1). Purposive sampling technique was used to sample 135 participants. A total of 12 FGDs were conducted with a range of 7 to 10 participants. The FGDs were held with youth (men only, women only, and mixed groups with a total of 4 FGDs for each group) who are involved in agribusiness in the urban and peri-urban areas of Lilongwe district. These youth include both those in ACADES and those who are not in ACADES but are involved in agribusiness in the two districts. ACADES focuses on employment creation and economic empowerment by redefining the agricultural sector, transforming it into a viable and desirable career for youth. ACADES aims to promote youth development in agribusiness through skills development, empowerment, capacity building, resource mobilisation, advocacy, and access to inputs and markets. The 17 KIIs were composed of local leaders, government officials from five government ministries (agriculture, youth, labour, education, and trade), officers from government and non-governmental institutions and youth bodies. These participants provided in-depth and detailed information about agribusiness and youth employment situations in the urban and peri-urban areas of Malawi.

The data collection process was reviewed and approved by IITA’s Internal Review Board (IRB); the approval number is IRB/IF-CA/008/2021.

### 2.2 Quantitative analysis: Construction of variables

One key step in the quantitative analysis was the construction of variables to use in the econometric analysis. In the quantitative analysis, youth employment in agribusiness is the main (dependent) variable which comprises on-farm and off-farm employment categories including wage and self-employment. With regards to the employment categories, the study distinguishes four types of employment in agribusiness: employment in farming involves a youth who works on a family farm or engages in ganyu (casual labour) on farms; it is labelled ‘*Farming*’. Employment in a mix of farming and off-farm non- agricultural wage activity involves a youth who is engaged in ‘*Farming*’ and self or wage employment in a non-agricultural sector; it is labelled as ‘*Farm_NoAgWage*’. Employment in an off-farm agricultural wage activity is defined as any work outside farming but in agriculture (self or wage employment); such form of employment can be alone or combined with other activities such as farming or a non-agricultural household business; it is labelled ‘AgWge_Farm_Biz’. Employment in a mix of Farming and non-agricultural business activities or apprenticeship is labelled as ‘*Farm_Biz_Skill’*. It involves a youth employed in ‘*Farming*’ but also apprenticeship or in non-agricultural household business activities such as home-based or outside home enterprises (not in agriculture).

In line with the literature review on the push and pull factors affecting employment [19, 21], the quantitative analysis retains exogenous variables that reflect demographic factors, institutional support (credit and extension services), asset ownership, market access and shocks. Specific demographic variables include age, sex, marital status (whether the youth is married, divorced/separated, widowed, or never married), religion (whether the youth have a religious belief or not) and household headship (whether the youth is household head or not). For household size, it consists of the number of family members while the dependency ratio reflects the number of dependents (individuals below the age of 14 years and above 64 years of age) per family. Additionally, education consisted of 4 levels: no formal education, primary, secondary and tertiary; the variable reflects the highest educational attainment for each respondent. The tertiary educational level includes a diploma, undergraduate degrees, or postgraduate qualification. The variable on access to credit reflects whether the youth has received credit or not (0 for no; 1 for yes). A similar code is used for access to agricultural extension services. The wealth index reflects the ownership of household and productive assets such as home appliances and agricultural tools. Other variables include idiosyncratic shocks (whether the household experienced an idiosyncratic shock or not), livestock ownership (tropical livestock unit owned), landholding (land measured in hectares), distance to the nearest tar road, and distance to the nearest market in kilometres.

### 2.3 Data analysis

#### 2.3.1 Quantitative analysis: Econometric models

This paper uses two logit regression models. The first model, a bivariate logit model, is used to estimate key factors determining urban and peri-urban youth employment in agribusiness. Here, youth employment in agribusiness is a binary indicator. The bivariate logit model is specified as follows:

$$Y_{i}=ln\left(\frac{P_{i}}{1-P_{i}}\right)$$ (1)

$$Y_{i}=ln\left(\frac{P_{i}}{1-P_{i}}\right)=\beta_{0}+\beta_{1}x_{i1}+\beta_{2}x_{i2}+\beta_{3}x_{i3}+\cdots +\beta_{1k}x_{ik}+\varepsilon_{i}$$ (2)

Where:

*Y_i_* = Dependent binary variable (youth ‘i’ is employed in agribusiness = 1, or unemployed = 0)

*P_i_* = Probability of youth ‘i’ being employed in agribusiness

1 – *P_i_* = Probability of youth ‘i’ being unemployed

*β*_0_–*β_ik_* = Regression coefficients

*x*_*i*1_ = Explanatory variables

The second model, Multinomial Logit Model (MNL) is used when there are more than two categories and the dependent variable is categorical [23]. The MNL is used to identify key determinants that affect engagement of urban and peri-urban youth into specific agribusiness employment categories. The MNL is specified as follows:

$${EMA}_{ij}=\beta_{0}+\beta_{1}x_{i1}+\beta_{2}x_{i2}+\cdots +\beta_{k}x_{ik}+\varepsilon_{i}$$ (3)

Where:

*EMA_ij_* = dependent categorical variable (youth ‘i’ is employed in agribusiness activity ‘j’ = 1, unemployed = 0)

*β*_0_ … *β_ik_* = Regression coefficients

The MNL uses the same explanatory variables used in the bivariate logit model (Eq 2). In MNL, one category of the dependent variable which has a large number of observations is chosen as the reference category [24]; in this case, ‘Unemployed’ was set as the reference category. The Relative Risk Ratio (RRR) were reported for the MNL, and the coefficients were computed in relation to the reference category.

The bivariate logit model was used to identify key determinants of youth employment in agribusiness, whereas the multivariate analysis provided more insights on the determinants affecting youth employment in specific categories of agribusiness employment. The econometric analysis was conducted in STATA 14.2 and sampling weights were used in the regression analysis to ensure that the results are nationally-representative.

#### 2.3.2 Qualitative analysis: Deductive coding approach

Qualitative data were analysed using deductive coding (thematic analysis) aided by NVivo software [25]. In the study, a deductive coding approach was undertaken in several stages, as suggested by Bernard [26]. Firstly, line-by-line coding of all text from field notes and interview transcripts from both FGDs and KIIs was done. Then, broad ideas and concepts identified were assigned codes to structure the data. The codes were then synthesized and iteratively grouped into relevant themes (predetermined categories or themes). The themes within the data were compared against one or more themes that are proposed in existing literature and compared across all cases. Then, the results were further synthesized into three broader themes with sub-themes nested within them. The data was coded into the following themes: (i) key factors that influence youth employment in agribusiness; (ii) opportunities faced in agribusiness; and (iii) challenges faced in agribusiness. The key questions asked were: what are the main factors that influence youth to be in agribusiness? How do these factors influence youth to be employed in agribusiness? What are the opportunities and challenges youth face in agribusiness, and how do they affect their employment in agribusiness? The study adopts the definition of youth by the African Union (AU) Commission, which defines youth as a person aged between 15 and 35 years.

## 3 Results

### 3.1 Quantitative analysis: Socio-economic characteristics of urban and peri-urban youth engaged in agribusiness in Malawi

In the urban and peri-urban areas of Malawi, the average age of the youth who were either unemployed or employed in agribusiness was 26 years in 2016–17, with 37.9% being males (**Table 1**). These youths stayed in households which comprised about 5 people on average and 24.4% of them were household heads; the others were related to the household head either as a spouse, a child, etc. Most of these urban and peri-urban youth (55.9%) had no formal education. About 29.1 percent had access to credit, while 55.3 percent had access to agricultural extension services. Less than half of these youth had experienced idiosyncratic shock; they also had an average landholding of about 0.02 hectares and an average livestock unit of 0.11. In addition, these youth lived about 1.45 km from a tarmac road and 1.57 km from the market (**Table 1**).

**Table 1:** Summary statistics for urban and peri-urban youth aged 15–34 years in Malawi.

Variable		Employed		Unemployed		Total	
		N = 3253		N = 4336		population: 2,763,712	
						N = 7589	
Age (years)		25.23	(0.17)	26.47	(0.16)	25.96	(0.15)***
Household head (%)		24.23	(0.01)	24.55	(0.01)	24.42	(0.01)
Sex (%)		42.90	(0.02)	34.48	(0.01)	37.92	(0.01)***
Marital Status (%)							
	Married	53.91	(0.02)	62.93	(0.02)	59.24	(0.02)***
	Separated/divorced	8.69	(0.01)	7.15	(0.01)	7.78	(0.01)*
	Widowed	1.15	(0.00)	1.62	(0.00)	1.43	(0.00)
	Never married	36.25	(0.02)	28.30	(0.02)	31.55	(0.01)***
Religion (%)		95.36	(0.01)	97.74	(0.00)	96.76	(0.00)***
Household size (people)		5.05	(0.07)	5.38	(0.08)	5.24	(0.07)***
Dependency ratio		0.95	(0.03)	1.04	(0.03)	1.00	(0.03)***
Education Level (%)							
	No Education	66.12	(0.02)	48.89	(0.02)	55.94	(0.02)***
	Primary	12.58	(0.01)	13.38	(0.01)	13.06	(0.01)
	Secondary	19.64	(0.01)	31.56	(0.02)	26.68	(0.01)***
	Tertiary	1.66	(0.00)	6.17	(0.01)	4.33	(0.01)***
Asset Index		0.09	(0.11)	0.72	(0.13)	0.47	(0.11)***
Extension Service (%)		72.69	(0.02)	43.30	(0.02)	55.32	(0.02)***
Credit (%)		30.99	(0.02)	27.82	(0.02)	29.12	(0.02)**
Shock (idiosyncratic) (%)		43.69	(0.02)	47.96	(0.02)	46.21	(0.01)***
Land ownership (ha)		0.04	(0.02)	0.01	(0.00)	0.02	(0.01)**
Livestock ownership (tlu)		0.16	(0.02)	0.08	(0.01)	0.11	(0.01)***
Distance to road (km)		1.72	(0.10)	1.26	(0.08)	1.45	(0.09)***
Distance to market (km)		1.40	(0.10)	1.68	(0.11)	1.57	(0.10)***

Note: Standard errors are given in parentheses; tlu = Tropical Livestock Unit; ha = hectares; km = kilometres.

*** p<0.01

** p<0.05

* p<0.1 denotes significant levels.

Source: Authors’ estimation based on IHS4 data.

There were key differences between the youth employed in agribusiness and the unemployed youth based on the Wald test (**Table 1**). There was a significant difference in terms of age: the youth employed in agribusiness were slightly younger (25.2 years) on average than the unemployed (26.5 years). About two-thirds of the youth employed in agribusiness were female (57.1%); yet, among the unemployed youth, the majority were also female (65.5%). More than half of the youth employed or unemployed were married. Also, the youth employed in agribusiness were less educated than the unemployed youth. More specifically, 66.1% of the youth employed in agribusiness had no formal education, compared to 48.9% of the unemployed youth. The average household size for the youth employed in agribusiness was slightly smaller than that of unemployed youth. Also, the average dependency ratio for the youth employed in agribusiness was 0.95, whereas it was higher, at 1.04 for the unemployed youth (**Table 1**).

There were also significant differences in terms of access to agricultural extension services and credit. For example, most of the youth employed in agribusiness (72.7%) had access to agricultural extension services compared to the unemployed youth (43.3%). In addition, the youth employed in agribusiness were more likely to have access to credit than their counterparts. The results also showed that the youth employed in agribusiness experienced slightly less idiosyncratic shock compared to the unemployed youth group. Additionally, the statistics show that the youth employed in agribusiness had an average landholding of 0.04 hectares, while the unemployed youth had an average of 0.01 hectares. Also, there was a significant difference in the number of livestock units owned. For instance, the youth employed in agribusiness held an average livestock unit of 0.16, while the unemployed youth held 0.08 units on average. The average distance to the road for the youth employed in agribusiness was higher than for the unemployed youth. However, the youth in agribusiness faced a shorter distance to market compared to the unemployed youth (**Table 1**).

### 3.2 Quantitative analysis: Distribution of urban and peri-urban youth by sex and agribusiness employment category

Out of the more than 2.5 million urban and peri-urban youth who were either unemployed or employed in agribusiness in Malawi in 2016/17, about 59.08 percent were unemployed, and 33.4 percent were employed solely in farming (**Table 2**). About 1.69 percent of these youth were receiving an agricultural wage or were combining the agricultural wage with other income sources such as farming or a household non-agricultural business. Of these, about 86% were engaged in agricultural wage work only. Approximately 4.23 percent of the youth were employed in a mix of farming and off-farm non-agricultural businesses, whereas 1.69% were involved in farming combined with a non-agricultural household business or apprenticeship (**Table 2**).

**Table 2:** Percentage of urban and peri-urban youth by employment category.

Employment Categories	Frequency	Percentage
Unemployed	1,632,801	59.08
Farming (family or ganyu)	923,080	33.40
Farming and off-farm non-agricultural wage	44,219	4.23
Farming and non-agricultural household business or apprenticeship	116,905	1.69
Off-farm agricultural wage alone or combined with either farming or non-agricultural household business*	46,707	1.60
Total population of unemployed and those employed in agri-business	2763712	100

**Source:** Author’s calculation based on IHS4 data

* the majority in this category (86%) are engaged in agricultural wage work alone

**Fig 2:**
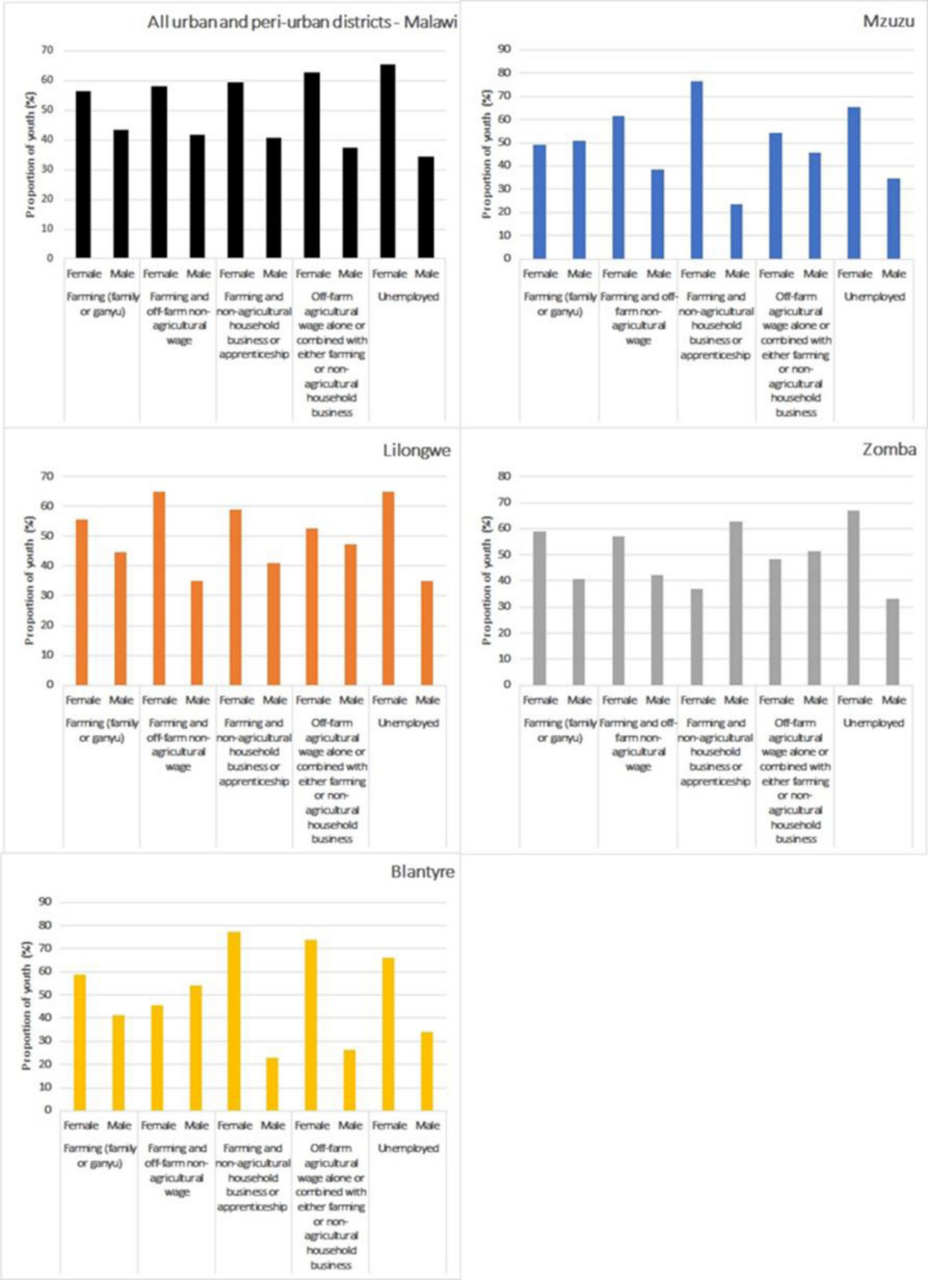
Gender distribution by employment category for urban and peri-urban youth-Malawi. **Source:** Authors’ computation.

Gender differences were observed in the employment categories (**Fig 2**). For one, the share of females among the unemployed youth was 66 percent. Women also outnumbered men across the categories of employment in agribusiness. Such a trend is a little nuanced when considering the different districts. For example, in Mzuzu, which is in northern Malawi, males slightly outnumbered women in the ‘farming’ employment category. For all other employment categories, women outnumbered men. In Lilongwe, which is in central Malawi, more females were employed compared to males in all employment categories. In Zomba, there were more males than females in the employment category where farming is combined with a business or apprenticeship and in the one involving off-farm agricultural wages. In Blantyre, which is in southern Malawi, females outnumbered males for all employment categories except the one involving farming combined with off-farm non-agricultural wages. More than 900 thousand (**Table 2**) urban and peri-urban youth in Malawi were engaged in farming only in 2016/17, and in this category, about 56% of them were female youth. The largest gender gap among the employed youth in Malawi was found in the employment category that consists of off-farm agricultural wages alone or combined with farming or a household business; examples of jobs in this category include clerks, beer processors, cooks, hotel employees, and maize millers. In this category, which had about 46,707 youth in 2016/17 (**Table 2**), 63 percent of them were female (**Fig 2**).

### 3.3 Determinants of youth employment in agribusiness

In this section, results are divided into three subsections. The first subsection presents results derived from both quantitative and qualitative approaches. The second part shows results derived only from the quantitative analysis, whereas the third part shows results derived only from the qualitative approach.

#### 3.3.1 Determinants of youth employment in agribusiness: Mixed-methods analysis

The determinants of youth employment in agribusiness that were identified through the quantitative and qualitative methods can be classified into the following themes: demographic factors such as sex (gender), marital status, and education; institutional support including credit access, access to agricultural extension services, and distance or access to markets; wealth (assets, livestock, and land ownership); and shocks.

On gender, the bivariate logit results showed that being male is associated with a higher likelihood of being employed in agribusiness compared to being female. More specifically, for the bivariate logit, the odds ratio on ‘sex’ is statistically significant and the value of the marginal effect is 0.061, meaning that being male instead of female increases the likelihood of being employed in agribusiness by 6.1% (**Table 3**). The results of the MNL show that male youth were more likely to be employed in ‘*farming*’ compared to female youth; for the other three employment categories, male youth were as likely as their female counterpart to be employed (**Table 3**). The results from the quantitative analysis are partly supported by the results from the qualitative analysis. More specifically, findings from all FGDs highlight that females tend to engage less in agribusiness (especially farming) than males because they face socio-cultural factors such as an increase in household and child-caring responsibilities as well as marital obligations that limit their engagement in agribusiness.

**Table 3:** Bivariate and multinomial logit analysis of determinants of youth employment in agribusiness.

Variable	Bivariate: employed vs. unemployed		Multivariate: employment in category of agribusiness activity versus unemployed							
			Farming		Farm_NoAgWage		Farm_Biz_Skill		AgWge_Farm_Biz	
	OR	ME	OR	ME	OR	ME	OR	ME	OR	ME
Age	1.022**	0.005	1.016*	0.002	1.052**	0.001	1.035*	0.001	1.056	0.001
	(0.009)		(0.009)		(0.026)		(0.019)		(0.037)	
Male	1.344***	0.061	1.421***	0.062	1.511	0.005	1.268	0.004	0.693	-0.007
	(0.119)		(0.136)		(0.464)		(0.256)		(0.220)	
Household head	0.738***	-0.063	0.728**	-0.053	0.569*	-0.007	0.785	-0.004	0.903	0.000
	(0.083)		(0.090)		(0.175)		(0.182)		(0.295)	
Married	0.570***	-0.116	0.592***	-0.087	1.033	0.004	0.643**	-0.009	0.322***	-0.015
	(0.065)		(0.074)		(0.329)		(0.140)		(0.114)	
Separated/divorced	1.066	0.013	1.192	0.037	2.582*	0.014	0.810	-0.011	0.247**	-0.022
	(0.188)		(0.226)		(1.298)		(0.279)		(0.172)	
Widowed	0.600*	-0.106	0.657	0.014	3.065	0.028	0.348	-0.023	0.000***	0.000
	(0.172)		(0.224)		(2.473)		(0.232)		(0.000)	
Religion	0.772	-0.054	0.748*	-0.059	3.276	0.019	1.007	0.004	0.671	-0.005
	(0.126)		(0.121)		(2.671)		(0.426)		(0.415)	
Primary education	0.820*	-0.043	0.727**	-0.072	1.714	0.008	1.230	0.016	1.641	0.007
	(0.097)		(0.091)		(0.607)		(0.337)		(0.626)	
Secondary education	0.640***	-0.094	0.517***	-0.129	2.360**	0.018	0.712*	-0.005	2.511**	0.017
	(0.057)		(0.050)		(0.849)		(0.135)		(1.037)	
Tertiary education	0.376***	-0.194	0.278***	-0.210	1.751	0.015	0.090***	-0.038	1.985	0.015
	(0.074)		(0.071)		(0.765)		(0.057)		(0.965)	
Household size	0.866***	-0.030	0.891***	-0.016	0.780***	-0.003	0.835***	-0.005	0.655***	-0.006
	(0.019)		(0.022)		(0.058)		(0.046)		(0.048)	
Dependency ratio	0.749***	-0.060	0.748***	-0.048	0.644**	-0.005	0.827	-0.002	0.719	-0.004
	(0.038)		(0.045)		(0.130)		(0.110)		(0.146)	
Access to credit	1.145**	0.028	1.127	0.016	1.105	0.001	1.488**	0.014	0.905	-0.002
	(0.079)		(0.085)		(0.264)		(0.239)		(0.223)	
Access to extension	2.929***	0.222	3.216***	0.198	3.286***	0.012	3.073***	0.026	0.632	-0.013
	(0.235)		(0.286)		(1.032)		(0.541)		(0.199)	
Land ownership (ha)	1.179*	0.034	1.245**	0.150	0.000***	-0.308	0.398	-0.026	2.088	0.019
	(0.111)		(0.136)		(0.000)		(0.262)		(1.776)	
Livestock (tlu)	1.332***	0.059	1.405***	0.062	0.738	-0.006	1.335**	0.007	0.644	-0.008
	(0.085)		(0.093)		(0.267)		(0.177)		(0.333)	
Asset index	0.856***	-0.032	0.827***	-0.035	0.944	0.000	0.956	0.001	0.954	0.000
	(0.030)		(0.032)		(0.087)		(0.057)		(0.055)	
Idiosyncratic shock	0.925	-0.016	0.854**	-0.034	1.133	0.002	1.150	0.008	1.366	0.005
	(0.052)		(0.053)		(0.238)		(0.175)		(0.356)	
Distance to road (km)	1.005*	0.001	1.007**	0.002	0.971*	0.000	0.985*	-0.001	1.001	0.000
	(0.002)		(0.003)		(0.016)		(0.009)		(0.004)	
Distance to market (km)	0.995	-0.001	0.995	-0.001	0.999	0.000	0.991	0.000	0.993	0.000
	(0.003)		(0.004)		(0.012)		(0.006)		(0.011)	
Constant	1.249		0.987		0.004***		0.069***		0.163*	
	(0.288)		(0.240)		(0.004)		(0.039)		(0.171)	

**Note**: Observations = 7312. Population: 2,651,929. The base category is unemployed. For categorical explanatory variables, the base case for marital status is “never married”; for education is “no education”. Standard errors are in parentheses. Regression is corrected with sampling weights.

*** p<0.01

** p<0.05

* p<0.1 denotes statistically significant levels.

The bivariate logit results showed that being a married or widowed youth was associated with a lower likelihood of being employed in agribusiness than being single (never married) (**Table 3**). The MNL results revealed that being married decreased the likelihood of being employed in farming (-8.7%); in a mix of farming and a business or apprenticeship (-0.9%); or in agricultural wage work either conducted alone or in combination with farming or a business (-1.5%). For the latter employment category, being separated also decreased the odds of employment compared to being single (never married). The findings from all mixed FGDs revealed that marital status influences youth to be in agribusiness, especially for females. Some married males were engaged in agribusiness but also tried to work in non-agricultural jobs or businesses in search of more remunerative opportunities and income. Participants underlined that married females tend to engage less in agribusiness because of their husband’s jealousy that they will indulge in immoral conduct with their male counterparts (**Table 3**).

As expected, findings from the bivariate logit model revealed that more educated urban and peri-urban youth had a higher likelihood of not being employed in agribusiness (**Table 3**). The odds ratios for the variables on education were statistically significant, and the values for marginal effects related to these variables decreased with the education level, going from -4.3% for primary education to -19.4% for tertiary education. The MNL results show a similar trend for employment in farming: less educated youth have a higher likelihood of being employed in farming. However, for two of the other agribusiness employment categories that involve agricultural or non-agricultural wage work, youth who had completed secondary education were more likely to be employed in these other categories compared to uneducated youth (**Table 3**). Such results are not surprising, as young people engaged in farming can receive training from their parents and community for farming; however, their engagement in other wage work usually requires a higher educational attainment. Findings from all FGDs confirmed that education is an important determinant of youth being employed in agribusiness, as it equips youth with skills and knowledge in agribusiness. However, all FGDs and key informant interview participants highlighted that the formal education system is more theoretical and that additional practical training in agribusiness would be needed. In all FGDs with non-ACADES participants, it was found that youth lack access to agribusiness education (training). Moreover, in all focus group discussions, participants revealed that most of the youth have little know-how in agribusiness activities, and the education curriculum does not focus much on agribusiness with a practical approach. Particularly, participants from ACADES underscored that the training programs provided by ACADES are very helpful, but more can be done. Attesting to that, a government official pointed out that:

“*Education programs do not focus much on agribusiness*, *despite it being a sector that has the potential to provide vast socio-economic benefits*. *Adding to that*, *education mostly focuses on a theoretical approach*. *This is a problem as youth lack the practical know-how in agribusiness activities*”. (Male key informant, Ministry of Youth, December 2019, Lilongwe)

Another government official reiterated that:

“*Education is mostly class oriented*. *Despite the education system putting in place entrepreneurship programs*, *Malawian culture does not promote entrepreneurship but white-collar jobs*. *The education system is creating robots as it does not harness creativity and critical thinking; this is killing opportunities for youth*”. (Male key informant, National Youth Council of Malawi, December 2019, Lilongwe)

The combination of the quantitative and qualitative results on education imply that ‘lack of education’ is a push factor for youth’s engagement in ‘farming’ and educational opportunities are a pull factor for the youth involved in other agribusiness employment categories.

The bivariate results show that having access to credit increases the likelihood of a youth being employed in agribusiness by 2.8%. However, the MNL results show that credit access is a positive determinant only for the youth engaged in a mix of farming and business or farming and apprenticeship. For all other employment categories, including sole farming, credit access seems to have no impact on engagement (**Table 3**). Access to credit is the most important determinant influencing youth to be employed in agribusiness, as it enables youth to have financial capital. In mostly all ACADES and non-ACADES FGDs, participants narrated that youth find it difficult to acquire credit, especially from banks, as they do not have the collateral banks require. However, female youth FGDs highlighted that female youth are sidelined in accessing credit compared to male youth. Here, we can conclude that the quantitative results reflect that most youth in agribusiness had very limited credit access in 2016/17 (**Table 1**); the qualitative analysis reveals the aspirations of urban and peri-urban youth who express the importance of credit access in enabling success in agribusiness.

Based on the bivariate logit results, youth who received agricultural extension services were 22.2% more likely to be employed in agribusiness compared to those who did not receive such services (**Table 3**). Compared to all other exogenous variables, ‘access to agricultural extension services’ has the highest positive impact on the likelihood of being employed in agribusiness. Results from the MNL model also showed a positive relationship between access to agricultural extension services and the likelihood of being employed in all employment categories except the one involving agricultural work wages. In addition, ‘access to agricultural extension services’ was also the variable with the highest positive impact on the likelihood of employment in ‘*farming*’ and ‘*Farm_Biz_Skill*’. It was revealed during all FGDs that access to agricultural extension services is an important determinant of youth employment in agribusiness as it develops capacity to do agribusiness activities. Yet, youth face constraints related to access to agricultural extension services, such as having little or no access to agricultural extension services. Non-ACADES participants particularly indicated that public agricultural extension services mostly focus on older farmers, and young farmers are usually left out. One male youth highlighted that there aren’t enough and sometimes no face-to-face meetings with extension agents from both government and development partners.

Results on land ownership in **Table 3** show that an increase of one hectare in land size is associated with an increase of 3.4% in the likelihood of being employed in agribusiness. However, the MNL results showed that land ownership seems positively linked with the likelihood of being employed in ‘*farming*’ only; for all other forms of agribusiness employment, land access has either a nil or negative impact on the likelihood of engagement. However, findings from all FGDs and KIIs revealed that land is an important factor that influences youth in agribusiness. Limited access to land was highlighted as one of the major challenges the youth face in all FGDs. Youth in other forms of agribusiness other than farming pointed out that a lack of land makes them venture into off-farm agribusiness employment. Male FGD participants further explained that most youth do not own land; for those who do, the land size is less than one hectare and is usually still owned and used by their parents. In all female FGDs, it was narrated that it is more difficult for female youth to access land because of cultural views and inheritance practices that, in most cases, allow only men to own land. This hinders females’ investment in agribusiness, as they have limited access to land for production.

Results from the bivariate logit showed that an increase of one Tropical Livestock Unit (tlu) is associated with an increase of 5.9% in the likelihood of a youth being employed in agribusiness (**Table 3**). The results from the MNL model showed a positive relationship between livestock ownership and engagement in ‘f*arming*’ only or engagement in a mix of farming with a business or apprenticeship (Farm_Biz_Skill). For the agribusiness employment categories that involved agricultural or non-agricultural wage work, livestock ownership had no impact on the likelihood of engagement. FGD participants from all mixed groups underlined that having livestock is an important determinant for youth to be employed in agribusiness, as youth can be able to sell the livestock or livestock products. For example, youth with chickens can sell the eggs or the chickens. This enables youth to start a business because it acts as a starter pack for being in agribusiness.

Contrary to expectation, experiencing idiosyncratic shock seems to have no impact on the likelihood of a youth being employed in agribusiness; the odds ratio related to the variable ‘idiosyncratic shock’ is not statistically significant (**Table 3**). The result from the MNL model showed that experiencing an idiosyncratic shock reduced the likelihood of a youth being engaged in sole ‘*farming*’ by about 3.4%; however, for all other agribusiness employment categories, experiencing an idiosyncratic shock has no impact on the likelihood of engagement. The FGDs with mixed groups revealed that the presence of shock, such as the death or illness of family members, influences youth to be in agribusiness to support the sick family member or the family after the death of the breadwinner. Furthermore, participants highlighted that poor weather conditions (such as drought and erratic rainfall) negatively affect their engagement in agribusiness, especially farming.

Results from the bivariate logit model showed that an increase of one kilometre in the distance to a tarmac road would lead to an increase of 0.1% in the likelihood of the youth being engaged in agribusiness. From the MNL model, a positive and significant relationship between the distance to the nearest tarmac road and the likelihood of engagement is also found for the youth engaged in ‘f*arming*’. Such result reflects that the youth who are currently engaged in ‘*farming*’ tend to be located further away from tarmac roads compared to unemployed youth; such youth are likely working in rural areas in Malawi with limited access to tarmac roads. FGD’s findings revealed that long distances to the road as well as poor road conditions negatively affect youth engagement in agribusiness. These conditions push the youth out of farming as they increase the cost of transportation, thereby making the youth invest less in agribusiness, which decreases production. Thus, ‘better access to roads’ pulls the youth to engage in a combination of farming and a business or apprenticeship.

Based on the bivariate logit results, long distances to the market had no statistically significant association with a youth being employed in agribusiness. Moreover, results from the MNL model showed that long distances to the market had no impact on the likelihood of youth’s engagement in different agribusiness categories. Findings from all the FGDs and KIIs showed that access to the market (such as access to quality inputs, consistent buyers, and stable market prices provided through contracts) is an important determinant for youth to be employed in agribusiness. This enables youth to buy farm inputs (seed, fertiliser, etc.) from the market and sell their products at the right time and price.

#### 3.3.2 Determinants of youth employment in agribusiness: Quantitative analysis

In this section, determinants that were derived from the quantitative analysis but were not captured through the qualitative analysis are presented. One such determinants is age, and the results show that ‘older’ youth tend to engage more in agribusiness. More specifically, the results from the bivariate logit in **Table 3** show that an increase in the age of youth increases the likelihood of being employed in agribusiness. Similarly, MNL results revealed that the age of youth positively influenced youth employment in three of the four agribusiness employment categories; it’s only for the category involving some agricultural wage work that there seems to be no relationship between age and the likelihood of engagement (**Table 3**).

Results from the bivariate logit model also show that youth who are household heads are 6.3% less likely to be employed in agribusiness compared to youth who are related to a household head (**Table 3**). The results from the MNL model showed a similar result for the youth engaged in ‘*farming*’ only. Such results suggest that urban and peri-urban youth who are household heads do not consider ‘*farming*’ only as a viable source of livelihood. (**Table 3**).

The bivariate logit results show that an increase of one unit in household size would reduce the likelihood of a youth being employed in agribusiness by about 3%. The results from the MNL model also reveal a negative relationship between household size and the likelihood of engagement in any of the agribusiness employment categories. Such results show that agribusiness loses its viability as a source of livelihood for the youth, as their family size increases (**Table 3**).

Similarly, the results from the bivariate model show that an increase of one unit in the dependency ratio is associated with a decrease of 6% in the likelihood of youth being engaged in agribusiness. Such a tendency is also observed for youth engagement in ‘farming’ only. Here, we can conclude that sole ‘farming’ loses its viability as a source of livelihood with an increase in the number of dependents in a household (**Table 3**).

#### 3.3.3 Key challenges and opportunities for youth’s engagement in agribusiness: A qualitative approach

In this section, we present results that were derived from the qualitative analysis but not captured through the quantitative analysis. These results focus mainly on the key challenges and opportunities related to youth employment in agribusiness.

***Opportunities*.** The qualitative analysis revealed that agribusiness value addition and integration into value chains provide youth with opportunities to find employment. All FGD and KII participants underscored that opportunities exist in food production, processing, and marketing services that youth can engage in. Participants, particularly from ACADES, highlighted that the availability of programs that support youth with training, access to inputs, credit, and markets helps provide youth with the opportunity to engage in agribusiness. On the other hand, non-ACADES participants indicated that such programs that support youth in agribusiness are helpful but do not reach all youth in Malawi. One female youth indicated that agribusiness programs have the potential to offer youth training in agribusiness and access to credit, inputs, and markets through which they can gain skills and employment. But the programs need to be gender sensitive. Additionally, one government official from the Ministry of Agriculture pointed out that:

“*Agribusiness is a hot issue and has the potential to provide opportunities for all youth in Malawi*. *However*, *the agribusiness sector needs a structure that is creative and inclusive of youth through technology-driven loan acquisition*, *and a conducive policy environment*, *as these will excite youth to get into agribusiness and be able to experience the full agribusiness opportunities”*. *(Male key informant*, *December 2019*, *Lilongwe)*

Nevertheless, it was revealed during all FGDs that the opportunities in agribusiness for youth are still few and not yet realised because of the lack of government investment in the sector and in youth. Therefore, this discourages youth from considering employment in agribusiness, as they opt for employment in other sectors. As such, adequate support and investment by the government and development partners are needed in the agribusiness sector to create more opportunities for youth.

***Challenges*.** The results from FGD participants identified a range of challenges that youth face in agribusiness that affect their engagement. Participants ranked the challenges faced in agribusiness (**Table 4)** and highlighted the major challenges.

**Table 4:** Challenges faced by youth in agribusiness.

Challenges	Ranked
Lack of access to credit	1
Limited access to improved farm inputs	1
Inadequate education (training) in agribusiness	2
Lack of potential land for production	3
Lack of access to markets	3
Limited access to extension services	4
Poor weather conditions	4
Inadequate youth agribusiness programs	5

**Source:** Focus Group Discussions, 2019

The findings from all FGD participants ranked lack of access to credit and lack of access to farm inputs as the first major challenges youth face (**Table 4**). Participants revealed that urban and peri-urban youth were unable to access credit facilities due to a lack of collateral, which hinders agribusiness activities and, as a result, negatively affects engagement in agribusiness. Participants in all FGDs pointed out that the complex loan acquisition procedures with high interest rates preclude them from acquiring a loan, which hampers engagement and the success of agribusiness enterprises. The ACADES official elaborated:

“*All youth in Malawi (both urban and rural youth) are regarded as risky clients due to a lack of collateral and viewed as not serious*. *Credit facilities need to view all youth as potential clients so as to cater for these age groups in order for them to engage in agribusiness”*. *(Male key informant*, *December 2019*, *Lilongwe)*

An official from the Clinton Development Initiative explained that:

“*Policy rates need to be brought down to improve access to credit among youth in agribusiness*, *both in urban and rural areas*.*” (Male key informant*, *November 2019*, *Lilongwe)*.

Similarly, all FGD participants revealed that limited access to improved farm inputs such as seeds, fertiliser, and farm equipment is due to a lack of money to buy the inputs, which is a challenge youth face in agribusiness (**Table 4**). Participants in all focus group discussions underlined that youth are less likely to use improved inputs, which affects engagement in agribusiness. Moreover, participants from ACADES underscored that youth have little access to inputs and delayed delivery of these inputs, which affects the growth of agribusiness enterprises. In addition, inadequate education in agribusiness was ranked as the second major challenge youth face, which affects engagement in agribusiness. All focus group discussion participants elaborated that most youth have little knowledge of agribusiness, affecting the growth of agribusiness or employment in agribusiness.

Moreover, lack of access to fertile land for production and limited access to markets were ranked as the third major challenges youth face in agribusiness (**Table 4**). Land is an essential asset in agriculture. However, participants in all FGDs revealed that potential land for production is scarce, with much of the land being privately owned or owned by parents, who utilise the land, which affects the ability of the youth to engage in agribusiness. In addition, findings revealed that limited access to markets for their products hinders youth from selling their products, which further affects engagement in and growth of an agribusiness enterprise. All FGD participants highlighted that unstable market prices and a lack of consistent buyers of their products leads to them being exploited by unscrupulous traders (vendors) who buy products from the youth by setting their own prices. This interference deters youth’s activities and engagement in agribusiness. Participants in ACADES FGDs underscored that despite ACADES providing market access, sometimes (more specifically, once a year or every two years), there is a delay in buyers purchasing their products; this makes it difficult for them to sell their produce on time. Non-ACADES participants narrated that there are limited or no buyers for their products, which makes youth sell their products to dishonest vendors. An official from the Ministry of Trade said that:

“*Agriculture and agribusiness are wealth*, *and therefore*, *market systems should be in place to support youth in the agribusiness sector for easy trade*. *Because if there is no trade*, *there is no economic growth”*. *(Interview with a male key informant*, *November 2019*, *Lilongwe)*

The ACADES official narrated that:

“*The market is messed up*, *and there is a lack of market information*. *But the market is large*, *and youth*, *given the right resources and proper market system*, *are able to produce products that Malawi imports from other countries*. *(Male key informant*, *December 2019*, *Lilongwe)*

Similarly, a youth leader from Mhub added that “*market accessibility is hard for most youth in terms of inadequate market access*, *limited market information and market infrastructure which hinders youth’s engagement in agribusiness*”.

The fourth challenge was limited access to agricultural extension services and poor weather conditions (**Table 4**). In all FGDs, participants emphasised that youth lack access to agricultural extension services. Participants underscored that agricultural extension services are non-existent with inadequate extension services from both the government and development partners. Nevertheless, despite Lilongwe University of Agriculture and Natural Resources (LUANAR) providing agricultural extension education, a lot needs to be done to reach most youth in agribusiness in Malawi. Furthermore, all FGD participants revealed that poor weather conditions affect the ability to obtain high-quality produce in large quantities as they decrease plant populations and distort crop development. Participants underscored that such an effect on the produce makes it less marketable due to poor quality and quantity. In all FGDs, participants emphasised the need for improved farm equipment, such as irrigation solar pumps, to help youth produce without hurdles.

In her remarks, an agricultural extension officer elaborated:

“*Extension workers are few with one extension worker working with 2000 farm households*. *Adding that they mostly worked with already organized and established farmers and mostly youth are left or few are in those groups*. *This affects the level of youth engagement in agribusiness*, *as extension workers help increase youth adoption of new farm practices”*.

The fifth challenge was inadequate agribusiness programs for the youth (**Table 4**). Participants in all mixed groups underlined that agribusiness programs for the youth are scarce, with the few existing programs not fully addressing the bottlenecks related to access to markets, farm inputs, land, and credit. Participants in all FGDs pointed out that youth’s access to agribusiness programs can help provide youth with training, inputs, loans, and other resources needed for agribusiness engagement and growth. Both ACADES and non-ACADES participants viewed ACADES as a good initiative that is helping youth in agribusiness in terms of access to training, inputs, markets, and market information.

#### 3.3.4 Key challenges and opportunities for youth’s engagement in agribusiness: Qualitative versus quantitative approach

Based on the bivariate logit model, the number one factor that enhances youth’s engagement in agribusiness is ‘access to extension services’ (**Table 5**). That variable has the highest marginal effect with a value of 0.222, and this implies that having access to extension services increases by 22.2% the likelihood of an urban or peri-urban youth being engaged in agribusiness. The variable with the next highest statistically significant effect on the likelihood of being engaged in agribusiness is “tertiary education” with a value of -0.194. An urban or peri-urban youth with tertiary education is 19.4% less likely to get engaged in agribusiness; such a result suggests that the lack of education is a negative factor that pushes youth into agribusiness. The next key factor affecting the likelihood of a youth being engaged in agribusiness is related to marital status. More specifically, being married or widowed reduces the likelihood of getting engaged in agribusiness. This result suggests that being married or widowed hinders youth’s engagement in agribusiness.

**Table 5:** Key determinants identified through the quantitative analysis.

Variable	Bivariate: agribusiness. vs. unemployed		Multivariate: employment in category of agribusiness activity versus unemployed							
	Agribusiness	Rank	Farming	Rank	Farm_NoAgWage	Rank	Farm_Biz_Skill	Rank	AgWage_Farm_Biz	Rank
Access to extension services	0.222	1	0.198	2	0.012	4	0.026	2	NA	NA
Tertiary education	-0.194	2	-0.210	1	NA	NA	-0.038	1	NA	NA
Married	-0.116	3	-0.087	5	NA	NA	-0.009	4	-0.015	3
Widowed	-0.106	4	NA	NA	NA	NA	NA	NA	0.000	5
Secondary education	-0.094	5	-0.129	4	0.018	2	-0.005	6	0.017	2
Household head	-0.063	6	-0.053	10	NA	NA	NA	NA	NA	NA
Male	0.061	7	0.062	8	NA	NA	NA	NA	NA	NA
Dependency ratio	-0.060	8	-0.048	11	-0.005	6	NA	NA	NA	NA
Livestock (tlu)	0.059	9	0.062	7	NA	NA	0.007	5	NA	NA
Primary education	-0.043	10	-0.072	6	NA	NA	NA	NA	NA	NA
Land ownership (ha)	0.034	11	0.150	3	-0.308	1	NA	NA	NA	NA
Asset index	-0.032	12	-0.035	12	NA	NA	NA	NA	NA	NA
Household size	-0.030	13	-0.016	14	-0.003	7	-0.005	7	-0.006	4
Access to credit	0.028	14	NA	NA	NA	NA	NA	NA	NA	NA
Age	0.005	15	0.002	15	0.001	8	0.001	8	NA	NA
Distance to road (km)	0.001	16	0.002	16	0.000	9	-0.001	9	NA	NA
Household head	NA	NA	NA	NA	-0.007	5	NA	NA	NA	NA
Separated/divorced	NA	NA	NA	NA	0.014	3	NA	NA	NA	NA
Access to credit	NA	NA	NA	NA	NA	NA	0.014	3	NA	NA
Separated/divorced	NA	NA	NA	NA	NA	NA	NA	NA	-0.022	1

Source: Authors’ computations using inputs from Table 3

A comparison between the ranking of key determinants based on the quantitative analysis (bivariate analysis) (**Table 5**) and the ranking of constraints based on the qualitative analysis (**Table 4**) shows that access to extension services is common to both rankings. Hence, access to extension services can be considered a key factor that would enable urban and peri-urban youth in Malawi to successfully engage in agribusiness. Education, and more specifically, adequate training in agribusiness, is another factor that would help the youth successfully engage in agribusiness. Lack of training in agribusiness is identified as the second most important constraint to the engagement of youth in agribusiness based on the qualitative analysis (**Table 4**). However, in the quantitative analysis, education, which mainly consists of secondary education, has been identified as a key determinant that helps the youth branch out of sole farming and engage in additional activities such as wage employment.

Lack of access to productive land is another key constraint to youth’s engagement in agribusiness, based on the qualitative analysis (**Table 4**). However, for the quantitative analysis, the size of cultivated land is a key determinant that enables youth to engage in sole farming (**Table 5**). For the youth who combine farming with additional activities, land access is not a key enabler. This result might reflect the fact that the youth who branch out of sole farming conduct farming activities on land owned by others.

Based on the qualitative analysis (**Table 4**), credit access is a key determinant affecting youth’s engagement in agribusiness. From the quantitative analysis, credit access is a key enabler for youth’s engagement in agribusiness, only for those who combine farming with a business or apprenticeship (**Table 5**). Such results reflect that such youth are financially literate and are hence more likely to successfully use credit.

Lack of access to markets (due to inadequate markets, marketing information and infrastructure) and a lack of improved farm inputs has been identified as a key constraint to youth engagement in agribusiness based on the qualitative analysis (**Table 4**). In the quantitative analysis, market access for both farm inputs and output sales are measured by the distance to a tarmac road and the distance to a market. These two variables are not identified as key determinants in the quantitative analysis (**Table 5**). Such discrepancy might stem from the fact that the variables used to define market access (for both inputs and outputs) in the quantitative analysis do not capture the meaning of market access, as identified through the qualitative analysis. Indeed, market access as revealed through the qualitative analysis, involves access to quality farm inputs, consistent buyers, and stable output prices. For urban and peri-urban youth, it’s very unlikely that distances to roads and markets also equate to enhanced access to improved farm inputs, access to consistent buyers, and stable prices from season to season.

## 4 Discussion

This study provides an analysis of the determinants of urban and peri-urban youth engagement in agribusiness in Malawi. A mixed-methods approach is used, which combines quantitative and qualitative approaches. The quantitative analysis is conducted on national-level data on urban and peri-urban youth in Malawi, and the qualitative analysis is conducted on data collected on the youth program, ACADES, and from decision-makers involved in youth engagement in Malawi.

Regarding land access, it is an important determinant of agribusiness employment for urban and peri-urban youth in Malawi, based on the mixed methods approach; however, previous studies have shown that youth usually have limited access to land [27–30]. Most youth face more constraints in accessing and using agricultural land compared to experienced farmers [31], and this limits semi-commercial and commercial production for the youth. As ascertained by Asfaw et al. [32], agricultural land accessed or used by the youth in Malawi is mostly less than one hectare on average, which makes it hard for profitable ventures.

In addition, the descriptive statistics showed that female youth in agribusiness outnumber males in almost all urban and peri-urban areas (**Fig 2**). This could be linked to the fact that women are traditionally involved in all aspects of farming, regardless of their location in Malawi; there is usually no gendered division of labour for agricultural activities in the country [33]. In Mzuzu, there were more male youths in ‘Farming’ compared to females (**Fig 2**). This result could be linked to patrilineal practices in northern Malawi (Mzuzu). In this region, women, unlike men, cannot inherit land. However, the central and southern parts of Malawi (Lilongwe, Zomba, and Blantyre) have matrilineal systems, and women inherit land from their mothers in these systems [33]. This explains why women outnumber men in Lilongwe, Blantyre, and Zomba for "farming.

The regression analysis also revealed that men are more likely to get employed in sole farming compared to women; however, men were as likely as women to get employed in other types of agribusinesses. Women represent a large percentage of the youth labor force in agriculture (**Table 1**). But they face many challenges in terms of limited access to resources, land, and credit facilities. Other studies have shown that women face low levels of education as well as gender norms that imply that they should be responsible for child and elderly care and domestic work [34, 35]. Young women also face gender challenges in agricultural value chains that affect their participation [36–38]. These constraints and gender norms limit the role of women in agriculture. An interesting result from the analysis is the negative association between non-single female youth and their employment in agribusiness (qualitative analysis). However, this could be linked to married female youth having marital obligations coupled with socio-cultural and gender norms (such as reproductive roles, caring for children, and domestic chores) embedded in the communities, which affect women’s time and engagement in agribusiness. Alternatively, it might be because of a lack of opportunities and resources, especially for widowed female youth. This inverse relationship has also been observed in previous studies that found that marriage reduced the likelihood for women to enter into employment in Malawi [19, 39].

Like many other studies [27, 40–42], this study shows that formal education negatively affects the likelihood of being engaged in agribusiness. The study also shows that less educated youth are more likely to be employed in sole farming, while more educated youth branch out of sole farming and seek other forms of employment, mostly off-farm employment. This is because off-farm employment usually requires one to be trained, unlike sole farming, which is usually taught by family members on-farm. Existing studies on the role of formal education in employment decisions found that formal education can help youth better understand and adopt agricultural innovations [30, 43]. However, this would call for skill development interventions that include technical and vocational training to ensure that the youth are skilled enough to adopt improved innovations and succeed in agribusiness [44].

Concerning credit, the analysis in this study shows that credit enables youth to acquire agricultural inputs and permits them to have the financial capital needed to start agribusiness activities. This result is consistent with previous studies that found that access to capital or credit was a key determinant of youth’ engagement in agribusiness in Kenya and Nigeria [45, 46].

Moreover, findings from the quantitative analysis revealed that access to agricultural extension services was positively associated with being employed in the various agribusiness employment categories. This positive association likely stems from the essential and practical knowledge youth acquire through extension services. However, the negative association between youth employment in an off-farm agricultural activity (AgWge_Farm_Biz) and access to extension services could be linked to the fact that off-farm employment usually does not require direct access to agricultural extension services compared to on-farm activities. Other studies have highlighted that youth in Malawi have limited access to extension services, which hinders agricultural intensification and engagement [30, 28].

The study findings on livestock show that owning livestock is an important determinant of youth’s engagement in agribusiness. The findings from the quantitative analysis showed that owning livestock increases the likelihood of being employed in agribusiness, particularly in sole farming and in a mix of farming and non-agricultural businesses. This is consistent with results from the qualitative analysis, which implied that youth can use the proceeds from selling livestock or livestock products to start agribusiness enterprises. This result suggests that livestock acts as a valuable asset that can be used to diversify agribusiness income. The positive influence of livestock ownership on youth’s engagement in agribusiness was also highlighted in another study, which showed that livestock ownership is positively linked with the number of hours that youth allocate to agriculture in Malawi, Niger, Nigeria, Tanzania, Uganda, and Ethiopia [42]. Both the quantitative and qualitative findings reveal that idiosyncratic shocks affect the likelihood of youth being employed in agribusiness; more specifically, some shocks, including climatic shocks, tend to push the youth to abandon sole farming. A previous study has shown that shocks don’t influence off-farm employment for men and women in Malawi, except for death or illness in the household, which tends to push Malawian men towards off-farm employment [19].

Our findings demonstrate that access to roads and markets is an important positive determinant of youth employment in agribusiness. Youth can more easily participate in agricultural transformation in a conducive environment that includes better linkages with markets [47–49]. Our study shows that improving market accessibility for urban and peri-urban youth in agribusiness should translate into enhancing access to consistent buyers and ensuring that youth receive adequate and stable market prices for their products.

This study demonstrates the importance of mixed methods in assessing determinants of youth employment. The quantitative analysis alone is useful to identify key determinants based on the revealed employment behaviour of the youth. However, the qualitative analysis taps into youth’s aspirations and their own understanding of the challenges and opportunities linked to engagement in agribusiness. As such, the qualitative analysis provides context to enrich our understanding of the determinants affecting youth engagement and, hence, derive more appropriate policy recommendations.

## 5 Conclusions and policy implications

This study combined quantitative and qualitative methods to identify the key factors that influence urban and peri-urban youth employment in agribusiness in Malawi. The results show that urban and peri-urban youth in Malawi who are engaged in agribusiness are either employed in sole farming; agricultural wage work, or a combination of farming and off-farm activities. The majority are in sole farming; fewer, more educated youth branch out of sole farming to get employed in agricultural wage work or in activities that combine farming with wage work, a business or apprenticeship. For the youth engaged in sole farming, the results show that push factors consist of limited education. Pull factors consist of access to agricultural extension services, and ownership of land and livestock. Additional key determinants identified through the mixed methods approach consist of gender, marital status, shocks, credit access, and distance to market. For the youth who tend to branch out of sole farming, key pull factors which were identified consist of higher educational opportunities and access to agricultural extension services. The key policy recommendation from this study is that programs aimed at supporting the engagement of urban and peri-urban youth in agribusiness in Malawi should consider a variety of factors. However, the core of such programs should involve enhanced access to extension services and practical training in agribusiness. These two elements are the main pull/opportunity factors that would facilitate the successful engagement of youth in agribusiness. One key limitation of this study is that the qualitative study was conducted in Lilongwe only. It would have been preferable to include other urban and peri-urban areas of Malawi in the qualitative analysis to better capture youth’s perceptions across locations and tribes. The mixed methods approach has also revealed that future quantitative research targeting urban and peri-urban youth in agribusiness would benefit from including indicators that capture ‘output price stability’ and ‘buyer consistency’ to measure market access.

## Supporting information

S1 File (DOCX)

S2 File (ZIP)
