# Supplementary material for: Analysing urban and peri-urban youth employment in agribusiness in Malawi
Source: PLoS One. 2023 Sep 21;18(9):e0290877. doi: 10.1371/journal.pone.0290877 (PMC10513215; doi:10.1371/journal.pone.0290877)
Supplement: S1 File — (DOCX) [file pone.0290877.s001.docx]

**QUESTIONS**

| **Qu.#** | **KEY QUESTIONS** | **PROBING QUESTIONS** |
| --- | --- | --- |
| 1 | How is the youth unemployment problem in the area? | How it is like finding work in the area? |
| 2 | In which sectors do different categories of young people find work in? |  |
| 3 | How do different categories of youth (men, women etc.) earn money? | What differences exist between male and female in earning money? Please explain why |
| 4 | To what extent does education prepare youth for self-employment? Please explain | How effective is the education curriculum in stimulating youth employment? |
| 5 | In your opinion, what do you think about agribusiness in promoting youth employment? Please explain why |  |
| 6 | What are the main factors you consider that determine youth employment in agribusiness? | How do these factors enable youth employment in agribusiness? |
| 7 | What opportunities are there in agribusiness for youth? Please explain |  |
| 8 | In your opinion, how do you view ACADES in promoting youth employment? |  |
| 9 | How does ACADES initiatives provide youth with a good opportunity for employment? | How accessible are these initiatives to different categories of youth? |
| 10 | How is the engagement of females and males in these agribusiness initiatives? |  |
| 11a & 11b | How many agribusiness initiatives or programs are available in community that support youth? | How accessible are these programs or initiatives to men and women? |
|  |  | If there are no or limited programs, what sort of programs are most important for young people? |
| 12 | How far does youth employment in agricultural related enterprise contribute to well-being of youth? |  |
| 13 | What are the main challenges youth face in agribusiness? |  |
| 14 | What strategies do you think should be put in place to address challenges youth face in agribusiness? Please explain why | Which areas of support do youth require to run a successful agribusiness venture? |
| 15 | In your opinion, what strategies should government and development partners put in place in relation to youth employment in agribusiness? Please explain why |  |

**Lilongwe Msundwe ACADES youth groups**

Answers - Group1 - Lilongwe Msundwe ACADES (Mixed)

| Qu.# |  |
| --- | --- |
| 1 | it is very high as a lot of young people are not working due to lack of jobs. Majority find some earning through ganyu. It’s all about who you know to find a job. NGOs are far to find jobs; few cooperatives also offer jobs. Some jobs are there; just that youth are lazy |
| 2 | informal sector mostly farming i.e., livestock, builders, agribusiness. But there is no stable market and the ones there are far. |
| 3 | both males and females earn money through agribusiness and the earnings are the same because it just depends on how one thinks (mindset). As a group, no difference; but individually there are differences because of gender stereotypes. |
| 4 | Education helps one to: think well, gain skills, understand issue of farming, know difference between loss and profit, select good crops, find markets, remove ignorance, and think wisely. However, there is need to enhance practical approaches compared to theory, reduce school fees, introduce farming schools and more cooperatives. Changing of syllabus should stop unless it has more effective subjects; teaching English is also key |
| 5 | agribusiness would help young people with employment issues as it provides a potential for opportunities that youth can engage in |
| 6 | Lack of jobs; lack of basic needs; be independent because wealth is in land; learn from others; empower youth; reduce bad behaviours; reduce family problems and shock (death of family member); access to land, education; access to credit and markets. |
| 7 | It reduces lack of jobs due to its ability to provide different opportunities to youth, like crop production that leads to earning more income that helps meet basic needs, buy other farm inputs or livestock. Working in a group helps produce more, get loans easily and learn new things. |
| 8 | ACADES helps youth be independent and be able to provide employment for youth; through their programs, they support youth |
| 9 | training (learn good farming practices and farming different crops), input loans, find markets for youth |
| 10 | males engage more because they depend on themselves; females doubt themselves (how they have been brought up); females are busy with housework; land mostly belongs to males; culture also affects females’ engagement and gender in farming is a problem in the area due to lack of knowledge on it. |
| 11a&b | FAYE (but don’t really fulfil their plans). CICCOD (have many programs like education, health, agriculture, etc.) but ACADES is the biggest (however, they come late with seeds; they also have issues of market; project has good procedures; just a problem of membership fee) |
| 12 | Increase earnings; meet basic needs; reduce migration to other places; enhance farming land, farm productivity and budgeting |
| 13 | Poor timing on crop information; lack of markets; lack of capital; lack of farm inputs; little seed for farming; lack of training or education (practical); poor weather condition; lack of land; lack of proper roads to buy and sell produce, group disorganization; lack of storage facilities; crop diseases - low crop productivity |
| 14 | develop youth clubs (cooperatives); improve education system (practical approach); increase companies where youth can work, have fixed market prices, have youth Village Savings and Loans (VSLs), establish technical colleges that include aspects of agriculture and agribusiness. |
| 15 | Training and educating youth in agribusiness, having access to loans, lands, markets, access to inputs, improve road access in most areas, |

Answers – Group2 - Lilongwe Msundwe ACADES (Women)

| Qu.# | Answers |
| --- | --- |
| 1 | Youth unemployment is very high with most youth just staying home without anything to do. Most of them are women who tend to take care of kids or get into farming which is mostly causal labour |
| 2 | Most youth are into agriculture |
| 3 | females doubt themselves; males think females can’t do anything (undermined); parents favour males because they say females will marry someone else therefore, they (females) will be helped/supported from that side; loans are mostly given to males compared to females |
| 4 | It does not fully prepare youth for self -employment as it only focuses on white collar jobs. |
| 5 |  |
| 6 | Problems in homes (as husbands say fertilizer farming is for men); be independent; find money to meet basic needs; shock (death of family member or head which force youth to support their family) |
| 7 | find money to support the family through farming; learn more farming; meet basic needs; buy other farm inputs |
| 8 | ACADES helps provide youth with access to farm inputs, loans, training in agribusiness and link them to markets which promotes youth employment in agribusiness |
| 9 | ACADES initiatives provide youth with a good opportunity for employment through access to inputs, loans, training in agribusiness and access to markets. |
| 10 | The engagement of both female and male youth in agribusiness initiatives is high and equal. Both females and males participate equally |
| 11a | Just ACADES |
| 11b | Programs that support youth in access to credit, farm inputs and also training in agribusiness would be good for young people |
| 12 |  |
| 13 | Lack of access to farm inputs, training in agribusiness, lack of access to roads and markets. Limited access to credit facilities |
| 14 | Youth employment in agribusiness helps improve wellbeing of youth as it enables youth gain income for basic needs. |
| 15 | Improve access to farm inputs, credit facilities, enable access to land and markets for youth to buy and also sell their products. Support youth with training in agribusiness skills. |

Answers - Group3 - Lilongwe Msundwe ACADES (Men)

| Qu.# | Answers |
| --- | --- |
| 1 | Youth unemployment is bad with most youth lacking access to proper jobs |
| 2 | Youth in Malawi are mostly in the agriculture sector as it provides opportunities compared to other sectors |
| 3 | Males depend on themselves, but their females do not depend on themselves; they depend on others |
| 4 | Education does not fully prepare youth for agribusiness as it only focuses on the theoretical aspect and not the practical side. |
| 5 | Agriculture provides more employment opportunities through the opportunities in the value chain |
| 6 | be independent (household size is big); be knowledgeable about farming, because man is the engine at home; lack of jobs, experience some shock like death of the breadwinner |
| 7 | gain skills and knowledge in agribusiness; expand the business to other businesses in the value chain from production to sales |
| 8 | ACADES provides youth with access to farm inputs, trainings in agribusiness, access to markets and market information. |
| 9 |  |
| 10 |  |
| 11a |  |
| 11b |  |
| 12 | It helps provide youth with income for basic needs which helps improve the wellbeing. |
| 13 | Lack of access to adequate land for production, lack of farm inputs, roads, markets, lack access to loans, limited training on agribusiness, shocks such as poor weather conditions. |
| 14 | Provide youth with access to credit, land, farm inputs. Provide access to markets and agribusiness training. |
| 15 | Government and development partners should support youth in accessing loans, lands, farm inputs, training youth in agribusiness and providing them with opportunities that would enable them to get into agribusiness. |

**Lilongwe Msundwe youth groups not involved in ACADES**

Answers - Group1 - Lilongwe Msundwe youth group not involved in ACADES (Mixed)

| Qu.# | Answers |
| --- | --- |
| 1 | unemployment is high as most young people are not working due to lack of jobs |
| 2 | informal sector mostly ganyu |
| 3 | ganyu, farming and other small businesses. There is some difference as females work in the house mostly and they doubt themselves |
| 4 | education helps gain knowledge and skills that help youth in doing business undertakings; education enlightens. However, there is need for change like teaching agribusiness not just agriculture in schools; have a practical approach in schools, teach English (form 2 should come back) - syllabus keeps changing which is disturbing |
| 5 | it’s a very effective way to promote youth employment due to its promising areas and value to provide |
| 6 | lack of jobs; have little education; problems in the family like lack of money, death of family member, be independent; meet basic needs |
| 7 | buy livestock, fertilizer, and land from agribusiness sales; pay fees; find assets; meet basic needs; help the family and others; build house |
| 8 | From the way other youth have progressed, we believe it is helping youth in promoting employment. If ACADES would reach other youth like us, then it would help reduce the unemployment problem among the youth in the area |
| 9 | N/A |
| 10 | In agribusiness the engagement is the same just some difference since males can do more than females. And females spend more time taking care of children |
| 11a&b | only know ACADES that help youth in agribusiness |
| 12 | find money to meet basic needs, buy food, pay fees, acquire assets, and invest in other businesses, buy other farm inputs and all |
| 13 | lack of farm inputs (fertilizer, seed); unstable market prices; lack of markets; poor weather conditions; lack of land; lack of capital; diseases |
| 14 | develop an organized youth club (to easily get capital or loans); provide inputs; visit youth to support them (extension services) |
| 15 | Introduce technical colleges that focus on agribusiness; have programs that focus on education (training and agribusiness) |

Answers - Group2 - Lilongwe Msundwe youth group not involved in ACADES (Women)

| Qu.# | Answers |
| --- | --- |
| 1 | There no jobs for youth this makes youth unemployment rate to be very high. There are few opportunities that youth can engage in. |
| 2 | Agriculture sector is the most sector that has potential to provide employment opportunities. |
| 3 | There is a difference as mostly females are busy with housework but less difference in agribusiness when youth work in a group |
| 4 |  |
| 5 |  |
| 6 | Education, meet basic needs, having land, and livestock, experiencing family shocks like death, lack of money for food and fees. |
| 7 | buy livestock, have enough funds to cover basic needs like food clothes, obtain assets and pay school fees |
| 8 |  |
| 9 |  |
| 10 | Females doubt themselves and undermine each other. Females are busy at home and jobs are lacking for females |
| 11a&b | only ACADES |
| 12 | Improvement of living standards; buy land; improved health and able to have money for fees |
| 13 | Undermined by males; lack of land, markets, roads; lack of NGOs that support youth. Poor weather conditions, lack of access to loans, training in agribusiness. |
| 14 |  |
| 15 | Should have many NGOs supporting youth in agribusiness that will provide them with access to loans, inputs, link them to markets, enable youth to access land, provide youth with training or education in agribusiness. Government should ensure proper access to roads in most areas. |

Answers - Group3 - Lilongwe Msundwe youth group not involved in ACADES (Men)

| Qu.# | Answers |
| --- | --- |
| 1 | Youth unemployment is very high and most youth have nothing to do. |
| 2 | Most youth do ganyu (causal labour) in farms and some are builders. |
| 3 | not much difference in agribusiness but still males earn more than females because males work more |
| 4 |  |
| 5 |  |
| 6 | education (little or no education), marital status (had to support the family), household size to support family |
| 7 | buy other assets, able to educate children, improved living conditions |
| 8 |  |
| 9 |  |
| 10 | females engage less as they undermine themselves and doubt; they depend on men mostly (no gender balance really) |
| 11a |  |
| 11b |  |
| 12 | think well; budget well; improve social status; meet basic needs |
| 13 | lack of inputs including land, poor weather conditions, poor road networks, lack of markets, lack of access to loans. |
| 14 |  |
| 15 | government should support youth more, increase and improve markets and have stable prices; also, government should have more technical colleges that focus on agribusiness |

**Lilongwe Mitundu ACADES youth groups**

Answers - TIKONDANE YOUTH CLUB; Group1 - Lilongwe Mitundu ACADES (Mixed)

| Qu.# | Answers |
| --- | --- |
| 1 | Very hard - as it depends on the qualifications one has; hence only few people are employed as most youth drop out of school due to lack of fees. There is also nepotism (it’s all about connections). Another is due to lack of markets (selling products at low prices) |
| 2 | Most youth find work in agriculture through farming, working in tobacco estates or farms. Other find domestic work (shops, hospitals, or homes), kabaza (bicycle transport), minibus drivers or airtel agents |
| 3 | There is a difference because most females can do some jobs, but they undermine themselves. But in farming there is a balance; however, livestock farming is mostly done by males compared to females hence the difference in earnings |
| 4 | Education helps one to be knowledgeable but education in Malawi does not really help. What is needed is providing the skills or training in agribusiness and making markets available. The current educational system does not provide job opportunities. |
| 5 | It is good as it helps benefit the life of youth and prevent one from engaging in bad habits. It also helps expand or grow other businesses, buy livestock; it also helps create other jobs for other youth. Agribusiness is very effective in promoting employment |
| 6 | Capital - financial gain (income is more that capital); being in a group and doing business as a group made us to be in agribusiness. |
| 7 | because of the value chain there are a lot of opportunities; helps create other businesses; create self-employment and jobs for others; buy other resources like land, livestock (livestock also provides manure for crops) |
| 8 | it helps young people find employment due to their trainings, input loans and access to information and markets which helps to grow youths’ agribusinesses. This, in turn, creates self-employment and jobs for others in their farms and all. it helps young people be independent. |
| 9 | ACADES project provides training in agribusiness; gives loans; helps find markets, networking/access to information (share experiences, knowledge, and skills e.g., beans, making peanut butter etc.) |
| 10 | Males engage more than female, because of culture and married females are denied involvement by their husband due to jealousy and fear they would be involved with other men. Hence females fail to engage in agribusiness. (Marriage affects their engagement) - thus, there is need to change mindset of people (leaders) |
| 11a&b | not that many; only ACADES and Farmers Forum for trade and social justice- FFTSJ. But for FFTSJ, they (youth) did not continue as they were told to grown certain types of vegetable and bypass ACADES; FFTSJ also covered all age groups, unlike ACADES which targets youth. |
| 12 | have enough money to support the family; social status improves; find money for fees and hospital |
| 13 | Lack and delay of markets for other crops; poor weather conditions; lack of farming materials or/and inputs like solar pumps for watering the crops; hence youth have to farm smaller land size as they use water cans which is tiring to do (more especially for females); access to finance/capital (loans); lack of storage facilities; lack of more extension workers (mostly lack of coordination) |
| 14 | provide input loans; government and NGOs should work with ACADES to find markets abroad; agreement between community and NGOs to build warehouses; delays for markets should be reduced by engaging other NGOs |
| 15 | Find other markets (to have completion); training should be more frequent and on time; seeds to come in good time; provide loans for inputs like solar pumps to water crops; improve and increase extension services as they help very much and government should have strategies that are not linked to politics in the sense that most politicians just take advantage of people to win votes but don’t really help the people when they get the money. |

Answers - TIKONDANE YOUTH CLUB; Group2 - Lilongwe Mitundu ACADES (Women)

| Qu.# | Answers |
| --- | --- |
| 1 | it’s very high more especially for females due to the kind of jobs females can do; but unemployment is very high for both males and females |
| 2 | mostly work is in the informal sector mostly ganyu in terms of farming and domestic work |
| 3 | there is a difference because there are some jobs females find hard to do; but males can do them. |
| 4 |  |
| 5 | Agribusiness is a best thing that would help youth find employment in Malawi as is has so many things and areas in which one can do well. But the issue is that it lacks markets, training, loans, and extension services that would help boost agribusiness |
| 6 | Lack of jobs; be independent; improve income levels and living standards |
| 7 | improve basic needs and able to buy land due to the benefit of selling farm produce or seeds |
| 8 | it the best program that is supporting youth employment especially in agribusiness; young people tend to open other businesses through it and create employment for others |
| 9 | it provides training, input loans, access to markets and information |
| 10 | males engage more than females due to females being lazy to do some jobs. Husbands prevent females from joining these youth clubs because of jealousy. Land is hard to find; hence most men engage than women as they have better chances of getting land than women (culture and mindset). |
| 11 a&b | None; only ACADES |
| 12 | living standards at home and family improve; have more income to sustain their family/home |
| 13 | Access to finance; inputs (seed, fertilizer, farm materials); lack of markets |
| 14 | women should be given extra loans for them to engage more; providing loans on inputs; NGOs to come in to support youth, |
| 15 | Improve and increase access to markets; provide loans to youth |

**Answers - TIKONDANE YOUTH CLUB; Group3 - Lilongwe Mitundu ACADES (Men)**

| Qu.# | Answers |
| --- | --- |
| 1 | There is high unemployment rate in the area and Malawi in general, due to lack of job opportunities. |
| 2 | Ganyu mostly in farms; shops |
| 3 | Most females and males earn money through farming, usually doing causal labour or selling farm produce or products. |
| 4 | Education does not fully prepare youth for self-employment as it only aims to prepare youth for white collar jobs. |
| 5 | Agribusiness has very high potential in promoting youth employment in agribusiness through the opportunities the value chain has. |
| 6 | Access to markets, extension services and training in agribusiness; coming of ACADES through story of someone who was trained at Mlale and did well. Death of the family member that leads to employment in agribusiness (shock). Having access to road network in which youth can go buy and sell their agribusiness products which enables youth to be in agribusiness. Access to credit facilities. |
| 7 | Agribusiness has a lot of opportunities in which youth can find employment in production, processing, services. |
| 8 | ACADES promotes youth employment in agribusiness through the initiatives such as access to farm input loans, training in agribusiness, access to market information and market linkages. |
| 9 | ACADES provides training in agribusiness, access to farm inputs loans, linkages to markets and access to market information this helps provides employment opportunities |
| 10 | females engage less due their husbands being jealous; males engage more because they need to support the family (head of the house); mindset of some people who think it’s evil money (ignorance of community about agribusiness) |
| 11a | None; just ACADES |
| 11b | Programs that are and would be more important for youth will need to focus on training in agribusiness, providing youth with farm inputs and loans. |
| 12 | living standards at home and family improve; have more income to sustain their family/home; find money for fees and hospital |
| 13 | lack of finances; lack of farm materials or inputs; lack of markets, lack of access to roads, having shock in the family |
| 14 | need more seeds; improve and increase markets (stable market prices) |
| 15 | Reform the agribusiness policy and programs to empower youth in agribusiness, also the have more programs that support youth with training in agribusiness, provide access to credit facilities, access to markets. |

**Lilongwe Mitundu youth group not involved in ACADES**

Answers - TIYAMIKE YOUTH CLUB; Group1 - Lilongwe Mitundu youth group not involved in ACADES (Mixed)

| Qu.# | Answers |
| --- | --- |
| 1 | unemployment is very high. it is hard to find work especially in government due to nepotism (it’s all about who you know). As a result, youth are led to doing businesses; limited education also contributes to this. Nevertheless, there is lack of jobs. |
| 2 | both males and females find work in farming, but more men find jobs in other sectors like building roads or houses |
| 3 | not much difference; but women work more than men as they want to support their families |
| 4 | education doesn’t help much; it just helps remove ignorance. In the sense that education, can help one do business as they have some know-hows |
| 5 | it helps promote youth employment as one farms and sells the produce; tends to create self-employment and then employment for other youth who help/support in farms |
| 6 | Lack of jobs; be independent; improve income levels and living standards; pay fees, saw others succeed in agribusiness and the benefit they got. Having shock like death of family member(s), good or bad weather conditions may influence youth employment in agribusiness. Having access to agricultural extension service. |
| 7 | a lot; high income; good seed |
| 8 | N/A |
| 9 | N/A |
| 10 | there is 50/50 engagement between males and females in agribusiness |
| 11 a&b | none |
| 12 | It is slowly helping because we just started and haven’t seen the full benefits yet |
| 13 | lack of farm inputs (materials, fertilizers seeds); lack of markets and proper roads to the markets; lack of support from NGOs and government; lack of capital |
| 14 | Connect to fellow youth engaged with other NGOs; have more agricultural extension workers (mentors); NGOs should assist youth to be organized; have markets available as well as seeds |
| 15 | loans should be provided to youth more especially input loans; increase extension services and trainings in agribusiness/agriculture; there needs to be gender empowerment among the community (civic education); NGOs to support youth clubs in communities. make market available |

Answers - TIYAMIKE YOUTH CLUB; Group2 - Lilongwe Mitundu youth group not involved in ACADES (Women)

| Qu.# | Answer |
| --- | --- |
| 1 | very high |
| 2 | ganyu (farming) both males and females. Building/construction for males only |
| 3 | farming mainly; but there is lack of jobs; majority of them just stay home. |
| 4 | Education does help. However, many people finish school but lack jobs; hence education doesn’t really prepare youth for self-employment |
| 5 | it’s very effective in promoting employment among youth due to its potential in providing youth with many opportunities in the value chain but not yet tapped |
| 6 | Access to agricultural extension services, training in agribusiness, access to credit, land and farm inputs. Access to roads, markets and market information on agribusiness. And also, death of a member of the family can determine youth to be in agribusiness. |
| 7 | Youth can be involved in farming, production, processing, services/ marketing of the agribusiness products or produce. |
| 8 |  |
| 9 |  |
| 10 | 50/50 because of gender awareness by the community |
| 11a | None and there is need to have more youth programs that support youth in agribusiness by providing them with training in agribusiness, extension services, loans, inputs, linking youth to markets |
| 11b |  |
| 12 | It helps improve the wellbeing of youth through providing youth with income for basic needs |
| 13 | lack of extension services or officers; lack of land, inputs, markets |
| 14 | provide input loans to youth; make seed available; market accessibility and access to extension services and workers |
| 15 |  |

Answers - TIYAMIKE YOUTH CLUB; Group3 - Lilongwe Mitundu youth group not involved in ACADES (Men)

| Qu.# | Answers |
| --- | --- |
| 1 | Youth unemployment rate is very high with most youth just staying home. There is lack of jobs opportunities |
| 2 | Most youth are in Causal labour (ganyu) in farming and building/construction |
| 3 | 50/50, sell the same; small difference |
| 4 | Education helps one to be aware but not entirely; it also helps one be self employed |
| 5 | Agribusiness has a lot of areas such as production (farming), processing, services in which youth can find vast employment opportunities. Thus, agribusiness has the potential to promote youth employment in agribusiness |
| 6 | Youth employment in agribusiness improves livelihoods; good way to make money to meet basic needs, access to credit, inputs and markets determine youth to be in agribusiness. And also experiencing shock like death or illness of the family head or member can enable one to be in agribusiness. As well as weather conditions, access to proper road networks to the markets to buy and sell agribusiness products. Having access to agricultural extension service. |
| 7 | Youth can engage in farming, production, processing and/or services (marketing) in which they can gain employment and improve their lives. |
| 8 |  |
| 9 |  |
| 10 | 50/50 but mostly males due to the nature of work. Married females barely take part due to their husbands denying them participation; women also look down on themselves |
| 11 a&b | none |
| 12 | livelihoods improve |
| 13 | lack of markets; negative mindset of community about agribusiness and being in the youth clubs; lack of education/training |
| 14 | provide inputs, loans, more extension services/workers, more seed varieties |
| 15 | government to visit youth clubs to encourage and support youth together with local leaders. NGOs to also support youth through trainings, loans, information; NGOs should provide or have international markets. In addition, youth should be taught about value chains |

**KEY INFORMANT INTERVIEWS**

| **INTERVIEW QUESTIONS** |
| --- |
| 1. What do you think about the current youth employment situation in Malawi? |
| 1. To what extent does education prepare youth for self-employment |
| 1. What do you think of the current education curriculum in relation to preparing youth to work? |
| 1. What are the factors driving employment growth in agribusiness? |
| 1. How effective is agribusiness on promoting youth employment? |
| 1. What is the youth employment prospects from agribusiness? |
| 1. How many agribusiness initiatives or programs are available that supporting youth? |
| 1. How accessible are these initiatives or programs to youth? |
| 1. How far do these initiatives and programs support youth employment? |
| 1. How far does youth employment in agribusiness contribute to socio-economic well-being of youth? |
| 1. What can you day about the difference in the welfare of male and female youth in agribusiness? If any |
| 1. What contributes to these differences or lack thereof in socio-economic wellbeing among or between male and female youth? |
| 1. How can these differences if any be reduced? |
| 1. What policy instruments are available to development actors and policymakers in assisting formal and informal agricultural enterprises to create jobs? Why/why not. |
| 1. What are the underpinning issues or challenges that hinder successful operation of agricultural enterprises among youth? |
| 1. And are these issues or challenges the same for all youth in the country? |
| 1. How can challenges youth face in agricultural related enterprises be addressed? |
| 1. In your view, what policy recommendations should be adopted to solve youth employment problem? |

**Agro-processing – Youth-led enterprise. (**Male youth aged 32 years, Lives in Likuni, Lilongwe District. He is into farming and agro-processing of tomatoes, groundnuts, milk**)**

| Qu.# | Answers |
| --- | --- |
| 1 | It’s very bad - as the world recognizes few people and it’s mostly about connections (who knows who). It’s annoying. Most youth find work in the informal sector (mostly agriculture -ganyu) |
| 2 | Education is useless- need more practical work in education (practical approach to things will help young people). Also, there is a need to teach young people about money and entrepreneurship while still in school |
| 3 | Education in Malawi is not really preparing youth for self-employment only preparing youth for white collar jobs, which are also limited. There is need to reform the education curriculum to focus more on the practical aspect and more on agribusiness or entrepreneurship opportunities |
| 4 | Having access to land, farm inputs, credit facilities, having access to market and market information and training in agribusiness. |
| 5 | Agribusiness has big potential and opportunities been endless regarding the value chain this creates more opportunities for employment. |
| 6 | Having an enabling environment would influence someone to be in agribusiness, either more or less of something; exposure is another factor; and resources; technology and innovation. (When things are working, we do not need a policy).  Agribusiness is linked to every sector or/and thing. Helps create a lot of employment in value chains. |
| 7 | Initiatives: Mhub, Msika. However, the issue is money -funding is little. The programs however are not that accessible only because of social media - it requires one to be on social media. (Information is hard to find) |
| 8 | N/A |
| 9 | N/A |
| 10 | It improves livelihoods; creates a lot of employment. |
| 11 | There is difference in the wellbeing of youth. Though there is a 50/50 earning of money between different categories of youth involved in agribusiness compared to those not in agribusiness. And while females engage more than males because women want to help themselves. Agribusiness tends to contribute more the socio-economic wellbeing of male youth than female youth. |
| 12 | Male youth tend to have more opportunities than females which makes them gain more income thus improve their wellbeing. |
| 13 | Provide more opportunities to female youth so they can have the same advantage as male youth. |
| 14 | There are limited, there is need to invest in Agro processing; |
| 15 | Agro-processing industry is at infant stage which makes it a challenge. Packaging is also hard here in Malawi, especially Lilongwe; to get some packages, one must travel to Blantyre to get them. Attitude of Malawians – they believe Malawi products are inferior; lack of capital (funds); participation of vendors distorts the market. NGOs always hold back |
| 16 |  |
| 17 |  |
| 18 | Government and development partners should show farmers all value chains, so that they can choose which part of the value chain they want to supply with produce; improve market systems (linkages). NGOs should support more, where government has failed or is failing |

**MINISTRY OF LABOUR –** Female Government official from the Ministry of Labour in Malawi

| Qu.# | **Answers** |
| --- | --- |
| Q1. | - Bad, population is young, and the job market is not creating jobs in all levels. Access to employment is minimum both in private and public sector. |
| Q2 | - Informal economy – small piece work going on. Not a full-time job. There is a need. |
| Q3 | Moving from a system of trained of white-collar jobs will still take more time. Education system must start in primary school focusing on hands-on (practical skills) and a more focus on entrepreneurship in the colleges. |
| Q4 | - Growth in agribusiness is only possible through having access to loans, farm inputs, land, training in agribusiness, access to road networks, markets and market information, extension services. Also, death of the breadwinner can lead to youth being employed in agribusiness to support the family. Having good weather condition can lead to employment growth in agribusiness |
| Q5 | - Agribusiness is a way to go. The country is good but is not marrying everything together to achieve positive impact. Youth are best people to be tapped. There is need to explore other avenues not just tobacco. There is not much value addition. |
| Q6 | - Agribusiness helps or would help youth have different employment opportunities through the value chain. |
| Q7 | - Horticulture, training, animal farming. Can never go wrong with food. |
|  | - Youth employment has been talked about only 5 – 10 years. |
| Q8 | - Young people – due to lack of jobs. Passion. |
| Q9 | - Created jobs for others |
|  | - Challenge – market access |
|  | - Need to find ways to complete with other countries and find markets for producers in Malawi. |
| Q10 | - It helps youth acquire money for basic needs that helps improve their lives and wellbeing |
| Q11 | - Not much difference but just men tend to have more opportunities than females this maybe bring about some difference. |
| Q12 | - Access to finance support – support youth with collateral free loans (Bank programs to help youth) |
|  | - Cooperatives are not really working in Malawi |
| Q13 | - 50/50 |
| Q14 |  |
| Q15 | - Finance, market, information, businesses, training  - Politics (huge challenge), tribalism  - Not good at looking for markets (international relations)  - Transports was unstable (transport system) |
| Q16 |  |
| Q17 |  |
| Q18 | Deliberate policies that talk about entrepreneurship – Youth be given opportunity to be creative so that when finishing school, they know their passion. - Good documents (policies and laws) but implementation is bad. |

**ACADES –** Executive Director of ACADES, youth leader aged 32 years. (Farming, irrigation, and producing seeds, and providing loans to youth in terms of seeds, fertilizer etc)

| Qu.# | **Answers** |
| --- | --- |
| Q1. | - Youth unemployment is high. National Statistics Office (NSO) says it 23% but think it is very high than recorded. - It is hard to find employment, there is high population plus the industries in Malawi are growing at a low rate. - The government has been the main employer for the longest time but things have changed. Don’t have many NGOs/companies. - Skilled youth taking jobs for unskilled youth e.g., doing surveys jobs |
| Q2 | Most youth work in informal sector – like shops, hotels, farms doing ganyu (causal labour). But is usually male dominated |
| Q3 | Difference between rural and urban   - Small types of business (depending on economic activity of the area - Peace work in construction sites, farms (ganyu) which is seasonal. 80% of people have 1 acre of land. - Men earn more money than women. Work is physical, and cultural stereotypes in Malawi have defined jobs for woman and those for males e.g., gardening for male than females. |
| Q4 | Education does not prepare youth for self-employment. Education only fit the job market (white collar jobs)   - There is no course on how to register a company like business, marketing or enterprise - There is need to have critical courses relating to business - Soft skills that need to be offered in school - Agriculture subject really does not inspire one to see agriculture as a good or viable employment option - LUANAR curriculum was to primarily prepare and train extension workers to train farmers. |
| Q5 | Agribusiness has potential but has not been exploited yet.   - For example, exported products from other countries like peanut butter which Malawi can manage. - Production – scarcity of products like tomatoes, potatoes, onion can be expensive, - Huge opportunities in agribusiness (value chain) from production; agro-processing & value addition; trainings; technological advancement. - The market is large |
| Q6 | Access to information (extension or eduction)   - In Malawi, agriculture for food security not for business. - Most maize is produced but does not bring enough money - Don’t know how to grow high value crops like green pepper etc. - Agricultural sector is big in Malawi (interest in cash crops, education, extension services. - Need new agribusiness policies or strategies in Malawi   Access to resources (inputs and capital)  Market is messed up – lack market information  Most youth are not organized. They do not belong to any other group/cooperative, they usually work alone. (youth are risky clients and agribusiness is risky)  - Seasonal scarcity of agricultural products (production – farming)  - Agro procession and value addition – producing tomato sauce, jam, or mango juice and jam etc.  - Services – no consultant to help farmers set up fish farms (Its an innovate idea) |
| Q7 | - AICC – provide training - NYD (Network for Development) – skills development - Self-help Africa – capacity building - Comprehensive program/approach are mostly to youth. More NGO’s focus on 1 component like training. |
| Q8 | - Provide info on marketing - Working in groups (youth groups) - Organized youth - Provide inputs for production - Loan facility - Market facilitation   Sub-contract to youth after the get the grant – advocacy |
| Q9 | Easily accessible to all categories – open to all  But more males and females. Encourage more females to be part of the group, even in training and leadership 50/50.   - Women who are single or separated engage more but when they are married, they leave the groups. - Cultural issues   SAFI buys seeds but not sure when they buy |
| Q10 |  |
| Q11 | Any employment leads to well-being, economic well-being is a solution to most people, prevents bad behaviours/habits among youth, and able to access basic needs |
| Q12 | Males tend to have slightly more wellbeing than females because they tend to have more opportunities in agribusiness. |
| Q13 |  |
| Q14 |  |
| Q15 | Challenges   - No stable/sustainable market - Most NGOs focus on one component e.g., training only (lack simple initiatives) - Lack resources - Lack skills   Lack access to information on agriculture and markets |
| Q16 |  |
| Q17 | - Efforts to get youth organized - Deliberate policies with financial institutions in terms of interest rates, collateral (loan production have to be tailor made for youth and agriculture) - Strategies that ensure information is accessible by youth (have centres) - Need more training but focusing on business and financial literacy |
| Q18 | Government should focus more on policies to strengthen markets, skills development and resources.   - Market solution e.g., milk farming/ - AIP – Agricultural Investment plan (Agribusiness strategies) - Development partners should start helping youth - Promote economic empowerment and activities among youth which will help youth not engage in other bad habits   Note: most donors are focusing on the symptoms and not the cause  Have funding programs on youth economic empowerment |

**LUANAR –** University staff from the Department of Agribusiness and Agricultural Extension, Female aged 40.

| Qu.# | **Answers** |
| --- | --- |
| Q1. | There is high unemployment among youth. There is need to do a lot more investment in young people and agriculture |
| Q2 | Not a lot of youth in agribusiness especially youth in production. There are more in trading then production (informal economy) |
| Q3 | - LUANAR – enhancing agribusiness youth entrepreneurship mindset. (Since 2013 LUANAR established incubator)  - Revising education curriculum |
| Q4 |  |
| Q5 | Value chain – agribusiness is key option (Agricultural sector) |
| Q6 |  |
| Q7 | AYA; Jobs for youth; World bank, Farm concern (stakeholders to speak one language) |
| Q8 | Yes, some of the initiatives while limited access to most youth |
| Q9 | They help equip youth with some skills in agriculture and agribusiness |
| Q10 | To some extent they help contribute to socio-economic wellbeing of youth as youth have some income for their basic needs. |
| Q11 | No much difference as most youth lack the same resources, despite females tend to lack |
| Q12 |  |
| Q13 |  |
| Q14 | Policy is good, offers good guidance |
| Q15 | Lack of resources (inputs, finance/capital)  Lack of markets  Mindset of youth – attitude of youth |
| Q16 |  |
| Q17 | Create enabling environment – role of Government, academic and NGOs  Provide agribusiness resources to youth  Change mindset of youth – attitude of youth  Know-how, financing, legal aspects, networking, marketing  Education sector – should make youth see or think in a holistic way. (Need mindset change focusing on entrepreneurship) |
| Q18 | NAIP – National agricultural Investment Plan.  Coordinator is an issue. |

**MHUB –** Male youth leader at Mhub (focusing on youth, technology and incubator accelerator programs)

| Qu.# | **Answers** |
| --- | --- |
| Q1. | It’s a mess. No jobs we not creating jobs for youth.  Plan MGDs – no practically, no system, conducive environment policy |
| Q2 | Apart from government, even NGOs and banks.  Agribusiness |
| Q3 | More focus of papers and practical, don’t understand work environment.  But not on survival in the workplace  Introduction a program to prepare for work environment  Proper structure |
| Q4 | Education system - Overhold in many aspects – not dynamic not responding to situation in Malawi and the world.   - Skills taught are not really needed – there is need to review it often. - Specialization should be started early like secondary school |
| Q5 | - Has the potential to provide opportunity for youth. - Value addition part to absorb a lot of youth - Technology also helps in market - Potential to export |
| Q6 | Default thing cause economy is driven by agriculture (leaning on agriculture).  A bit of security despite being seasonal. Does not require |
| Q7 | ACADES (more like incubator) –  Land O Lakes (Msika)  Smoke free world  MHub – growth accelerator  ATI |
| Q8 |  |
| Q9 |  |
| Q10 | Agribusiness helps youth improve their socio-economic wellbeing through the income they make from production and sells. |
| Q11 |  |
| Q12 |  |
| Q13 |  |
| Q14 | Part of policies are good but not practical and enforced  NYP – 10% of government contract should go to youth. Youth are struggling  Harmonize policies as they do not speak to each other |
| Q15 | - Lack access to extension services - Lack of female special programs (international programs) - Interest rates are high - Mindset of youth - Lack of Access to land - Lack of access to markets - Lack of road to roads - Policy environment (proper policies are needed) - Incentives for people to start business - Value addition (taxes are too high) - Experiencing some shock in terms of death of family head or spouse - Peer (me too) – need creativity - Education in agribusiness (lack thereof) - Access to business development services |
| Q16 |  |
| Q17 | Need to recognize the youth in agribusiness and provide them with necessary tools, resources and room to do agribusiness activities. |
| Q18 | - Value addition concept – move into processing but should have a conducive environment - TEVETA misses a critical component in Malawi. Agribusiness should be topping in its program. TEVETA should recognize agribusiness. - Young people should recognize and go into business i.e., Small Medium Enterprises - Need more incubators in Malawi - Driven by entrepreneurs not academia - Linking technology to agriculture is better. Automize agriculture system. - Leverage on exponential growth of technology (technology can only make sense if we link it to agriculture. |

**BANKERS ASSOCIATION OF MALAWI –** The association official, Male aged 40.

| Qu.# | **Answers** |
| --- | --- |
| Q1. | There is high unemployment – not enough job opportunities |
| Q2 |  |
| Q3 |  |
| Q4 | Education does not really prepare youth for employment especially in agribusiness. |
| Q5 | Another opportunity /alternative |
| Q6 | - Access to finance is a limiting factor - Collateral - Risky consumer (youth) |
| Q7 | - NABWI - Working with ladies |
| Q8 |  |
| Q9 | In central region agribusiness is promoted because of the landscape |
| Q10 |  |
| Q11 |  |
| Q12 |  |
| Q13 |  |
| Q14 |  |
| Q15 | - Lack of access to Land - Limited access to finance (capital) - Banks are difficult - Microfinance is an alternative but as a cooperative is best |
| Q16 | Capital – to buy farm inputs as well as irrigation  Land access |
| Q17 |  |
| Q18 | Revamp schemes that were there during President Kamuzu time  Have deliberate policies for youth in agribusiness |

**LOCAL LEADER IN MITUNDU AREA-** Male Village Headman in Mitundu, Lilongwe District, Malawi.

| Qu.# | **Answers** |
| --- | --- |
| Q1. | It is very hard when they finish school to get employment, as there no jobs. |
| Q2 |  |
| Q3 | It’s hard because they do not finish school because of no fees and the schools are far. |
| Q4 | There are no programs that encourage youth in agribusiness |
| Q5 | Agribusiness should be promoted and be put in the law |
| Q6 | It’s beneficial (not really aware) |
| Q7 | Only one group of youth (40) |
| Q8 | Men (older) than young |
| Q9 | Agribusiness is effective and also help youth to find employment |
| Q10 |  |
| Q11 |  |
| Q12 |  |
| Q13 |  |
| Q14 |  |
| Q15 | - Lack of market - Lack of inputs - Lack of capital - Experience shock like death of breadwinner |
| Q16 | - Access to land - Access to inputs - Access to markets - Access to roads |
| Q17 | - Agreement - Networking - Extension services – (they are here are there) mostly available only to older men not young (Being together with young) |
| Q18 | Government should let extension officers support youth and educate them |

**LOCAL LEADER in Mitundu Area –** Male Local leader (Village group Head) in Mitundu.

| Qu.# | **Answers** |
| --- | --- |
| Q1. | It’s hard, one needs to have someone they know to find employment |
| Q2 |  |
| Q3 | School does not help – it also depends on knowing someone |
| Q4 |  |
| Q5 | Agribusiness is good, it offers the potential for employment. |
| Q6 |  |
| Q7 | ACADES |
| Q8 | - Mostly males than females - View of females being with males (perception) |
| Q9 |  |
| Q10 |  |
| Q11 |  |
| Q12 | Accessibility is okay! |
| Q13 |  |
| Q14 |  |
| Q15 | - Lack of markets - Lack of access to farm inputs (seeds and fertilizer) - Limited access to arable land |
| Q16 |  |
| Q17 |  |
| Q18 | - NGO should be doing things fast |

**EXTENSION OFFICER (AEDO) in Mitundu Area –** Female extension officer aged 33.

| Qu.# | **Answers** |
| --- | --- |
| Q1. | There few to no jobs which most youth can get into. The unemployment rate is high |
| Q2 | Education helpful but not really in Malawi because of how the education curriculum is structure. Does not focus more on the practical aspect of learning |
| Q3 | Education curriculum is more theory than practical. Needs to be reformed and improve course outlines |
| Q4 | Access to agricultural extension services and training in agribusiness  Access to land  Access to markets  Access to credit facilities |
| Q5 | Agribusiness has the ability to create more jobs than any sector and can help reduce the unemployment rate in Malawi, given that youth are provided the necessary resources. |
| Q6 | Youth can be employed or find employment from production, processing, to value addition, marketing and trading (services) |
| Q7 | Few that I know of mostly ACADES which helps youth with input loans and access to trainings in agribusiness. |
| Q8 | - ACADES is very accessible to youth but most youth don’t have the money to be a member hence hinders their participation but is an easily accessible program. |
| Q9 | - The provide youth the needed resources to start self-employment in agribusiness and also help create opportunities for other youth |
| Q10 | The wellbeing of youth are usually improved when they engage in any form of employment. Seemingly agribusiness, which provides youth vast opportunities to make money |
| Q11 | Its usually the same because of the benefits they get from agribusiness is somewhat the same. |
| Q12 |  |
| Q13 |  |
| Q14 |  |
| Q15 | - Inadequate extension workers (1 extension worker – 2000 farm households). Also encourage people even youth to be in a group (but youth are not organized people). - There is need to give youth a mother body to help them access extension services. - Extension services are now demand-driven |
| Q16 | - Most challenges are the same, but my vary in magnitude depending on the location and number of resources in the area. |
| Q17 |  |
| Q18 | Government and NGOs need to focus on increasing agricultural extension services especially among youth and also ensure they get the right training and knowledge in agribusiness |

**CLINTON FOUNDATION – Male official from Clinton Foundation - Agriculture Department**

| Qu.# | **Answers** |
| --- | --- |
| Q1. | Unemployment is very high in Malawi and lack employment opportunities |
| Q2 | Education system needs to be improved and reformed to accommodate changing employment landscape and skills needed for self-employment. |
| Q3 | Education only focuses on preparing youth for white collar jobs and not really entrepreneurship or business. Need more practical skills and trainings |
| Q4 | - Availability of farm inputs (seeds, fertilizer) - Access to loans/capital - Access to extension services - Stakeholder collaboration in promoting agribusiness |
| Q5 | Agribusiness is key in promoting employment among youth. It has potential to offer vast employment opportunities. |
| Q6 | Agribusiness has various prospects in value addition and agro-processing in which youth can have opportunities for employment. From production, processing, services/sales and marketing. |
| Q7 | There are some agribusiness initiatives that support youth but are not many which can target many youths. There is need to invest in such youth agribusiness programs. |
| Q8 | Yes, to some extent but still limited as most youth do not really venture in agribusiness due to lack of land, inputs and capital. |
| Q9 | - They provide youth with some small loans for inputs and secure or rent land. - Provide training and market information. |
| Q10 | Being employed in agribusiness helps youth have a source of income and also have food for their family, this help improve the socio-economic well-being of youth and their families. |
| Q11 | There is a slight much difference in terms of well-being of both male and female youth even though, male youth tend to have more than female youth. But female youth tend to farm more than males, hence having availability of food. |
| Q12 | - Male youth have more opportunities than female youth in terms of access to agribusiness opportunities thus the slight difference in their wellbeing. |
| Q13 | - Ensuring female youth are integrated and engaged in agribusiness programs and training. - Encourage females to engage in the programs and promote gender equally in the programs |
| Q14 |  |
| Q15 | - Lack of access to finance (capital)  - Lack of access to financial literacy education  - Lack of access to land for farming  - Lack of access to extension services |
| Q16 | - Some are the same but not all because of cultural differences and accessibility of resources. |
| Q17 | - Access to capital driven by policy rates needs to be brought down to improve access to credit among youth in agribusiness, both in urban and rural areas. |
| Q18 | Have deliberate youth in agribusiness policies that help promote youth employment in agribusiness. |
