# Supplementary material for: Analysing urban and peri-urban youth employment in agribusiness in Malawi
Source: PLoS One. 2023 Sep 21;18(9):e0290877. doi: 10.1371/journal.pone.0290877 (PMC10513215; doi:10.1371/journal.pone.0290877)
Supplement: S2 File — (ZIP) [file pone.0290877.s002.zip › IHS4_data/~WRL0240.tmp]

	(1)	(2)	(3)	(4)	(5)	(6)	(7)	(8)	(9)	(10)	(11)	(12)	(13)	(14)	(15)	(16)	
VARIABLES	coef	coef	coef	coef	coef	coef	coef	coef	coef	coef	coef	coef	coef	coef	coef	coef	
																	
1																	
hectares_measured	0.303**	(0.131)	-1.055*	(0.545)	-2,416	(107,209)	-2,340	(130,089)	-0.371	(2.647)	-4.200	(4.545)	-0.00121	(0.539)			
sex	-0.229***	(0.0525)	0.0571	(0.112)	-0.264	(0.431)	-1.044	(0.853)	-1.119*	(0.634)	-0.139	(0.209)	-0.180	(0.144)			
marriedstatus1	-0.612***	(0.0860)	-0.341*	(0.177)	0.664	(0.670)	-0.906	(1.449)	-0.414	(0.933)	-0.454	(0.277)	-0.192	(0.220)			
marriedstatus2	-0.246*	(0.126)	-0.427	(0.285)	0.272	(1.159)	-19.68	(43,716)	0.884	(1.236)	0.139	(0.474)	0.00265	(0.364)			
marriedstatus3	-0.507*	(0.289)	-0.537	(0.641)	-20.82	(104,806)	-20.12	(134,221)	-20.15	(105,457)	0.0967	(1.068)	-0.640	(1.046)			
o.marriedstatus4	-		-		-		-		-		-		-		-		
religions1	0.124	(0.163)	-0.155	(0.380)	-21.78	(68,664)	-20.38	(93,846)	-21.76	(77,601)	0.146	(0.745)	-1.436	(1.012)			
o.religions2	-		-		-		-		-		-		-		-		
edulevel1	1.282***	(0.204)	1.526***	(0.526)	0.696	(1.136)	-2.065	(1.623)	0.314	(1.234)	-0.690*	(0.408)	-0.390	(0.322)			
edulevel2	0.962***	(0.209)	1.435***	(0.532)	0.802	(1.155)	-22.71	(34,367)	-0.348	(1.334)	-0.123	(0.417)	-0.223	(0.337)			
edulevel3	0.686***	(0.202)	1.229**	(0.521)	-0.220	(1.129)	-1.092	(1.258)	-0.904	(1.208)	-0.00261	(0.336)	-0.0229	(0.291)			
o.edulevel4	-		-		-		-		-		-		-		-		
credit	0.0807	(0.0531)	0.369***	(0.107)	0.191	(0.439)	0.917	(0.782)	0.529	(0.563)	-0.217	(0.234)	0.368***	(0.142)			
extension	1.187***	(0.0532)	1.196***	(0.117)	0.545	(0.428)	0.500	(0.812)	0.670	(0.575)	-0.688***	(0.263)	0.946***	(0.147)			
shock_hh	-0.160***	(0.0497)	0.389***	(0.106)	-0.569	(0.426)	0.950	(0.860)	-0.240	(0.559)	0.243	(0.202)	0.0856	(0.139)			
age	0.0194***	(0.00656)	0.0190	(0.0135)	0.00117	(0.0513)	-0.0684	(0.112)	0.0307	(0.0694)	0.0564**	(0.0240)	0.0130	(0.0175)			
hsize	-0.0964***	(0.0151)	-0.144***	(0.0333)	-0.127	(0.140)	-0.0714	(0.220)	0.0236	(0.162)	-0.391***	(0.0689)	-0.201***	(0.0426)			
asset_indexx	-0.160***	(0.0159)	-0.0495	(0.0301)	-0.0453	(0.122)	-0.189	(0.213)	-0.223	(0.190)	0.0683	(0.0452)	0.0293	(0.0285)			
dependency_ratio	-0.276***	(0.0427)	-0.343***	(0.0982)	-0.839*	(0.475)	-0.709	(0.930)	-2.177***	(0.818)	-0.716***	(0.230)	-0.525***	(0.143)			
tlu	0.203***	(0.0425)	0.186**	(0.0801)	0.301	(0.199)	0.437	(0.334)	0.290	(0.238)	-1.015	(1.057)	0.101	(0.116)			
com_cd02a	0.00106	(0.000816)	-0.00274	(0.00274)	-0.0921*	(0.0534)	-0.194	(0.203)	-0.0845	(0.0716)	0.00209	(0.00179)	-0.0502***	(0.0142)			
com_cd18a	-0.00880***	(0.00230)	-0.0135**	(0.00547)	-0.00916	(0.0223)	-0.0303	(0.0455)	0.0287	(0.0221)	-0.0140	(0.0103)	-0.00512	(0.00723)			
2																	
3																	
4																	
5																	
6																	
7																	
8																	
o.hectares_measured															-		
o.sex															-		
o.marriedstatus1															-		
o.marriedstatus2															-		
o.marriedstatus3															-		
o.religions1															-		
o.edulevel1															-		
o.edulevel2															-		
o.edulevel3															-		
o.credit															-		
o.extension															-		
o.shock_hh															-		
o.age															-		
o.hsize															-		
o.asset_indexx															-		
o.dependency_ratio															-		
o.tlu															-		
o.com_cd02a															-		
o.com_cd18a															-		
o._cons															0	(0)	
Constant	-1.081***	(0.267)	-4.007***	(0.642)	-3.919**	(1.782)	-1.030	(3.380)	-3.695	(2.391)	-1.917**	(0.805)	-1.762***	(0.577)			
																	
Observations	9,049		9,049		9,049		9,049		9,049		9,049		9,049		9,049		
Standard errors in parentheses
*** p<0.01, ** p<0.05, * p<0.1
